# Managing arrhythmia in cardiac resynchronisation therapy

**DOI:** 10.3389/fcvm.2023.1211560

**Published:** 2023-08-07

**Authors:** Felicity de Vere, Nadeev Wijesuriya, Mark K. Elliott, Vishal Mehta, Sandra Howell, Martin Bishop, Marina Strocchi, Steven A. Niederer, Christopher A. Rinaldi

**Affiliations:** ^1^ School of Biomedical Engineering and Imaging Sciences, King’s College, London, United Kingdom; ^2^ Department of Cardiology, Guy’s and St Thomas’ NHS Foundation Trust, London, United Kingdom

**Keywords:** cardiac resynchronisation therapy (CRT), arrhythmia (any), atrial fibillation, ventricular arrhythmia (VAs), supraventricular arrhythmias, AF ablation, AV node ablation, heart failure

## Abstract

Arrhythmia is an extremely common finding in patients receiving cardiac resynchronisation therapy (CRT). Despite this, in the majority of randomised trials testing CRT efficacy, patients with a recent history of arrhythmia were excluded. Most of our knowledge into the management of arrhythmia in CRT is therefore based on arrhythmia trials in the heart failure (HF) population, rather than from trials dedicated to the CRT population. However, unique to CRT patients is the aim to reach as close to 100% biventricular pacing (BVP) as possible, with HF outcomes greatly influenced by relatively small changes in pacing percentage. Thus, in comparison to the average HF patient, there is an even greater incentive for controlling arrhythmia, to achieve minimal interference with the effective delivery of BVP. In this review, we examine both atrial and ventricular arrhythmias, addressing their impact on CRT, and discuss the available evidence regarding optimal arrhythmia management in this patient group. We review pharmacological and procedural-based approaches, and lastly explore novel ways of harnessing device data to guide treatment of arrhythmia in CRT.

## Introduction

It is well-established that clinical outcomes in heart failure (HF) patients receiving cardiac resynchronisation therapy (CRT) are significantly improved by maintaining biventricular pacing (BVP) burden over 90% (1). In patients receiving greater than 97% BVP, there is an even further reduction in HF hospitalisation and death (1). The most common reason for BVP% to be reduced is arrhythmia. In one retrospective analysis of over 80,000 patients, the most common reason for suboptimal BVP was atrial tachycardia (AT) or atrial fibrillation (AF), with other arrhythmic causes including non-AF/AT supraventricular tachycardia (SVT), premature ventricular contractions (PVCs), and non-sustained ventricular tachycardia (NSVT) (2).

Managing arrhythmia in CRT comes with unique challenges. Firstly, the reason for arrhythmia is often related to underlying substrate, including myocardial scar and cardiac chamber dilatation. This often makes the arrhythmia more challenging to treat, and thus ensues a vicious cycle whereby arrhythmia begets low BVP%, and low BVP% precludes effective cardiac remodelling. One such pathology is left atrial (LA) fibrosis, the presence of which both predicts a lower chance of success from AF ablation (3), and is more prevalent in those with reduced left ventricular (LV) ejection fraction (EF) vs. normal LV function (4).

In addition, BVP percentages reported by CRT devices are not wholly accurate in the presence of arrhythmia; although a device may state there is over 90% BVP, in those with permanent AF, a high proportion of this can often be in the form of fusion and pseudo-fusion beats rather than genuinely successful biventricular capture (5). Newer technology aims to overcome this over-estimation of BVP by detecting “*effective*” vs. “*ineffective*” BVP delivery (6), but this is not currently in place for the majority of CRT patients. Inaccurate BVP% reporting in AF patients may also go some way towards explaining the poorer outcomes demonstrated in CRT patients with AF vs. sinus rhythm, even when they have matching levels of reported BVP (7).

There are, however, benefits that arise from arrhythmia patients having CRT. Continuous rhythm monitoring by the CRT device has its clear advantages, leading to earlier arrhythmia detection and a richer source of patient data. Early detection allows for early intervention, which in AF, for example, has been shown to improve cardiovascular outcomes (8). In more recent years, device data has even been used to localise ventricular arrhythmias (VAs) and guide subsequent ablation treatment (9).

As well as providing data, there is also evidence to suggest that CRT itself has anti-arrhythmic properties, independently reducing the burden of arrhythmia in several studies (10, 11). In the case of VAs, the degree of protection offered by CRT appears to be related to the extent of LV reverse remodelling achieved. In one systematic review, when limiting analysis to 23 pure CRT trials (i.e., excluding trials with ICD-only treatment arms) with 6,455 participants, risk of VA was significantly reduced in CRT responders (with variable definitions of LVEF recovery) vs. non-responders (relative risk (RR): 0.46; 95% confidence interval (CI): 0.37–0.57, *p* < 0.0001) and even further reduced in CRT super-responders (defined as LVEF ≥50%) vs. those with LVEF <50% (RR: 0.22; 95% CI: 0.12–0.40, *p* < 0.0001) (12). Similar observations have been made regarding atrial arrhythmia burden and LA reverse remodelling after CRT implantation (13), further emphasising the complex interplay between CRT, heart failure and arrhythmia.

In this article we will review the impact and management of arrhythmia in CRT, discussing the available evidence, and highlighting areas where research is lacking. We will address atrial and ventricular arrhythmias in turn, before reviewing the impact of other HF medication in this population, and lastly explore how device data can be harnessed to specifically guide the management of arrhythmia in CRT.

## Atrial fibrillation

As well as being the most prevalent arrhythmia worldwide (14), AF is by far the most common arrhythmia in CRT, with a recent survey of over 11,000 CRT patients demonstrating an AF prevalence of 26% (15). We also know that the presence of AF is associated with a poorer response to CRT compared to sinus rhythm patients (16). Despite this, there is still a relative paucity of research dedicated to the management of AF in CRT patients. We are, however, able to infer some management strategies from those with HF with reduced EF (HFrEF), as this is by far the most common patient group treated with CRT (15).

### Rate vs. rhythm control in HFrEF

One of the main debates in the management of AF is regarding rate control [delivered through atrioventricular (AV) nodal blocking agents such as beta-blockers, or AV node ablation (AVNA)] vs. rhythm control [performed through the administration of antiarrhythmic drugs (AADs) such as amiodarone, AF catheter ablation or electrical cardioversion]. The original AFFIRM (17) trial in 2002 seemed to favour rate control, and when specifically investigating patients with reduced LVEF, Roy et al. (18) demonstrated no significant difference between the two strategies. However, in these early studies, rhythm control entailed the aggressive use of AADs and cardioversion, often with the cessation of anticoagulation upon restoring sinus rhythm. The inclusion of a relatively high number of participants in sinus rhythm at the point of randomisation in the AFFIRM trial (2,095/3,873; 54%) may have also diluted any comparable benefit from rhythm control in this cohort.

In more recent studies utilising *catheter ablation* as a rhythm control strategy, outcomes have been more favourable for rhythm control of AF in patients with HFrEF (19–21). Indeed, since the publication of AFFIRM, AF ablation techniques have continually improved with comparatively little change in medical management strategies. A recent meta-analysis comparing catheter ablation to medical therapy for AF in heart failure patients analysed data from eight trials between 2010 and 2022 (including the recently published RAFT-AF trial), with a total of 1,390 patients (22). 58% of patients had an LVEF ≤45%, with average LVEF ranging from 18% to 33% between included trials. Catheter ablation was found to result in significantly large reductions in both all-cause mortality (RR: 0.61; 95% CI: 0.44–0.84; *p* = 0.003) and heart failure hospitalisation (RR: 0.60; 95% CI: 0.49–0.74; *p* < 0.001) when compared to medical therapy, despite several included studies having nonsignificant outcomes in isolation.

**Figure 1:**
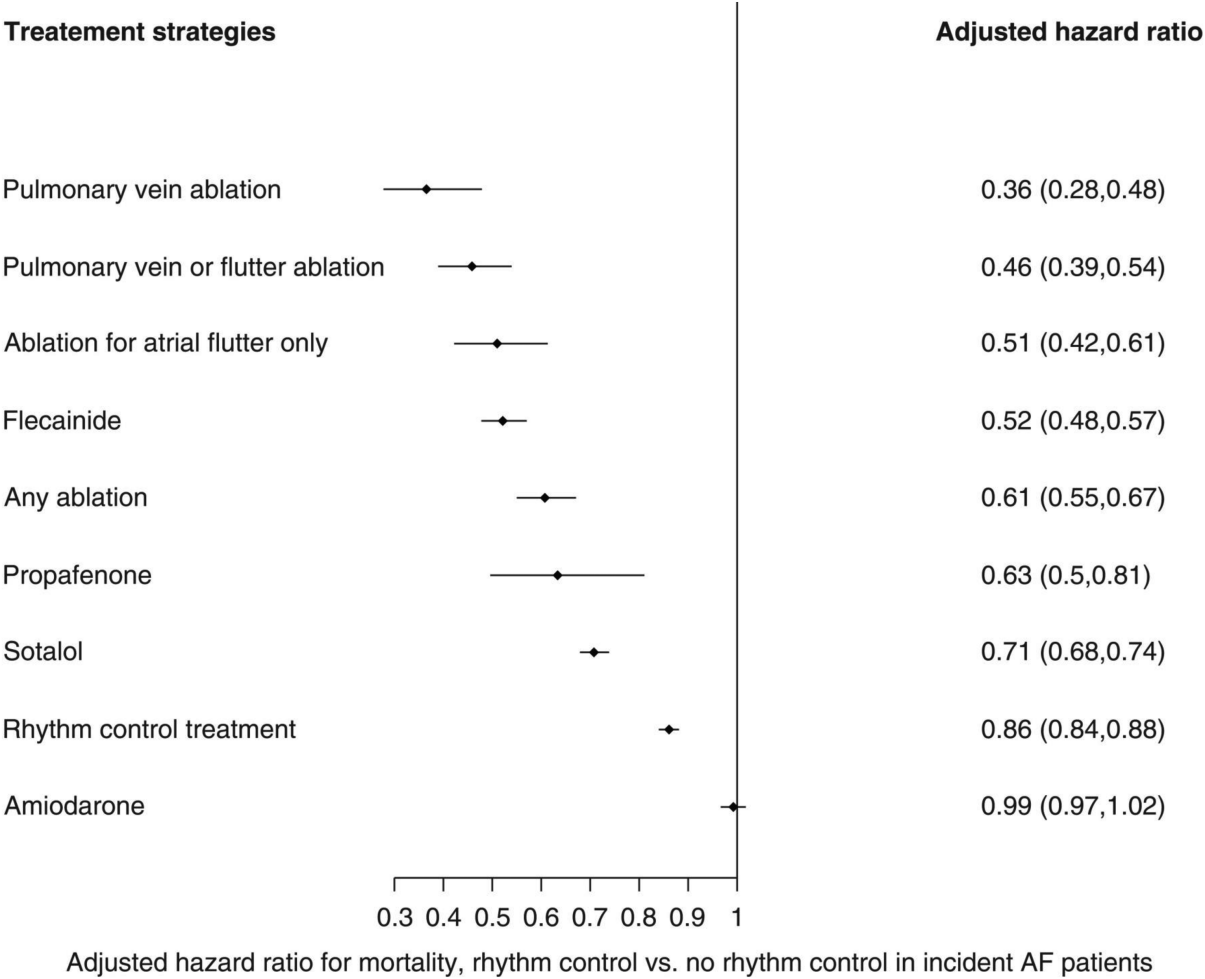
Multivariate-adjusted Cox regression analyses for mortality risk from rhythm control strategies when compared to no rhythm control. Reproduced with permission from Chung et al. (21).

In keeping with this is a recent population study by Chung et al. (21), where the health records of patients with new-onset AF (mean duration of 2.8 years from onset of AF to eventual treatment) were retrospectively analysed to compare outcomes in 28,497 who received rhythm control (including AADs and catheter ablation) vs. 196,676 who did not. In their multivariate-adjusted Cox regression analysis, adjusting for 25 variables including age and co-morbidities, pulmonary vein isolation (PVI) conferred the most survival benefit, with a two-third mortality reduction when compared to no rhythm control [hazard ratio (HR): 0.36; 95% CI: 0.28–0.48], followed by flecainide, propafenone, and sotalol (see Figure 1). A subgroup analysis of the 25,652 patients included with a “history of heart failure” (not further characterised) demonstrated similar benefit from a rhythm control strategy (HR: 0.73, 95% CI: 0.70–0.77) over no rhythm control, led by atrial flutter ablation (HR: 0.31, 95% CI: 0.2–0.49), pulmonary vein ablation (HR: 0.31, 95% CI: 0.14–0.69), and “any ablation” (HR: 0.46, 95% CI: 0.37–0.57) over all medical rhythm control options. Such observational data is clearly prone to selection bias, which may account for the relatively early divergence in their time-to-event curves. The low proportion of patients receiving rhythm control vs. no rhythm control also likely reflects the time period over which data were collected (1998–2016), as international guidelines have only more recently advocated the broader application of rhythm control for treating AF. Nonetheless, this study signals a benefit from rhythm control, especially in the form of ablation, in heart failure patients with relatively new-onset AF.

The only randomised controlled trial (RCT) specifically designed to assess mortality outcomes from AF ablation in HF patients with LVEF ≤35% is CASTLE-AF (19). This trial randomised HFrEF patients with paroxysmal or persistent AF to receive either optimal medical therapy (OMT) or AF ablation in addition to OMT. Of note, baseline medication usage was extremely similar between groups, including 32% in the ablation group and 31% in the OMT group taking an AAD, meaning this was more a study of *ablation* vs. *medication* rather than *rate* vs. *rhythm control*. Over a mean follow-up period of 37 months, significantly fewer patients in the ablation arm experienced the primary composite endpoint of death from any cause or heart failure hospitalisation (HR: 0.62; 95% CI: 0.43–0.87; *p* = 0.006) compared to those on medical therapy alone. For the 27.5% participants with a CRT-defibrillator (CRT-D), there was a nonsignificant trend towards a reduced risk of the primary outcome in those receiving ablation vs. medical therapy (HR: 0.54; 95% CI: 0.28–1.04). However, the applicability of these findings to a general HF population has been called into question (23); patient selection was slow and precise, with 87% of patients screened excluded from eventual participation, and 8 years taken to recruit 360 patients. As a result, the majority of those included were young men, with a median age of 64 years and 86% male participation. This is notable given women have consistently been shown to have poorer outcomes from AF ablation than men (24, 25) and the average age of initial HF diagnosis is nearer 74–77 years old (26, 27). When combined with the fact that only expert ablation centres took part in the trial, it is unclear whether CASTLE-AF's relatively low complication rates are truly representative of the average older HF patient undergoing AF ablation, especially in lower volume centres.

CASTLE-AF's findings are, however, consistent with several other RCTs investigating AF ablation vs. medical therapy in the HF population. Both the CAMTAF and CAMERA-MRI trials demonstrated a significant increase in LVEF at 6 months in HFrEF patients (mean baseline LVEF 35% and 33% and mean 6-month LVEF 39.9% and 50.1% respectively) when randomised to AF ablation vs. medical rate control, with CAMTAF also reporting significant improvements in functional capacity (*p* = 0.014) and Minnesota Living with HF Questionnaire (MLHFQ) scores (*p* < 0.001) (28, 29). Jones et al. found AF ablation was associated with improvements in peak oxygen consumption (VO_2_) (mean difference in VO_2_ max + 3.07 ml/kg/min; 95% CI: 0.56–5.59; *p* = 0.018), LVEF (mean difference + 5.6%; 95% CI: −0.1 to +11.3; *p* = 0.055) and MLHFQ scores (median difference −10.5, *p* = 0.019) at 1 year when compared to medical rate control in their RCT of 52 HF patients with LVEF ≤35% (30). Lastly, when compared to amiodarone, the AATAC trial found AF ablation reduced the risk of hospitalisation (RR: 0.55; 95% CI: 0.39–0.76; *p* < 0.001) and mortality (RR: 0.44; 95% CI: −0.20 to 0.96; *p* = 0.037) in CRT and ICD patients with LVEF <40%, with additional significant improvements in LVEF (*p* = 0.02) and MLHFQ scores (*p* = 0.04) at 2 years for those randomised to ablation (31). Interestingly in this particular trial, the average duration of AF prior to enrolment was extremely short at 8.6 ± 3.2 months [mean ± standard deviation (SD)] in the ablation group and 8.4 ± 4.1 months in the amiodarone group, compared to mean durations of 21–24 months in the aforementioned studies (28–30). This was perhaps due to the presence of a device, which could facilitate earlier arrhythmia detection. Overall, despite some variation in the specific method of ablation adopted, these trials consistently demonstrate superior LV remodelling, exercise capacity and quality of life outcomes from catheter ablation when compared to medical rate or rhythm control in the management of AF in HFrEF patients.

The decision whether to adopt a rate or rhythm control strategy is also influenced by whether the AF is *paroxysmal* and *persistent*, as catheter ablation success rates in the HF population vary significantly between these subtypes. One international multicentre registry of 1,273 patients found the long-term success of AF ablation was significantly lower in those with HF (defined as LVEF ≤45%) vs. those without HF if AF was persistent (57.3 vs. 75.8%, *p* < 0.001), but not significantly different if AF was paroxysmal (78.7 vs. 85.7%, *p* = 0.186) (32). Thus, catheter ablation is more likely to be successful if AF is paroxysmal rather than persistent, and this difference is amplified in those with HF. This likely relates to how early in the disease process AF has been diagnosed, with paroxysmal AF usually occurring many months-years before more persistent forms. Indeed, one meta-analysis of 4,950 patients undergoing AF ablation demonstrated a 27% lower chance of AF recurrence post-ablation if diagnosis-to-ablation time was below 1 year (33). The large EAST-AFNET 4 RCT demonstrated a mortality benefit from this approach, with early rhythm control (either in the form of AF ablation or AADs) within 1 year of AF diagnosis leading to significant reductions in cardiovascular death and stroke (8). Although the majority of EAST-AFNET 4's patients had HF with preserved ejection fraction (LVEF >50%), those in the lower LVEF categories of <40% and <35% trended towards greater benefit from early rhythm control than their preserved EF counterparts. However, due to small sample sizes, these trends were not statistically significant and should therefore be interpreted with relative caution.

Overall, these studies indicate that a rhythm control strategy (especially delivered through catheter ablation) may benefit HF patients even more than non-HF patients when compared to a medication-based rate control approach, particularly when for early, paroxysmal forms of AF vs. more long-standing, persistent varieties. Although we can infer some overlap between the HF and CRT populations, having a CRT device alters the AF ‘rate vs. rhythm control’ debate for several reasons. Firstly, compared to the average HF patient, HF-CRT patients are a specific subset of the HF population with the most severe levels of LV systolic dysfunction (LVEF ≤35%), associated with higher burdens of myocardial scar (34), and the administration of advanced HF drug therapy. These characteristics each have their own independent influence on arrhythmia burden and recurrence rates, altering the landscape of any rhythm control strategy in this cohort. Secondly, having a pacing device allows for comparatively more aggressive rate control, including the option of AVNA, which shifts the debate significantly. Nonetheless, given the relatively few trials specifically investigating rate vs. rhythm control in CRT, reviewing the corresponding evidence in HF populations provides a strong foundation when considering how to manage AF in CRT patients.

### Rhythm control in CRT

As mentioned, research into the management of AF in CRT patients is sparse, especially with regards to rhythm control. One small study randomised 43 CRT patients with AF to receive either rhythm control in the form of external electrical cardioversion, or rate control in the form of medication or AVNA as required (35). All patients received amiodarone. Both strategies significantly improved BVP from baseline, but the difference between groups at 1 year follow-up was not significant. LVEF, however, significantly improved in the rhythm control group, with a greater mean LVEF at 1 year compared to the rate control group (36.8% vs. 29.9% respectively, *p* = 0.039). With only 42% of rhythm control patients in sinus rhythm at 1-year, external cardioversion was not a particularly successful antiarrhythmic therapy. This makes us consider using AF catheter ablation instead as our rhythm control strategy in CRT, which we have already shown to improve outcomes when compared to medical rate control in the HF population (21, 28–30).

One small study by Fink et al. recruited 38 CRT non-responders to undergo AF ablation (36). Non-response to CRT was defined as either reduced BVP <95% due to AF, <1 point improvement in New York Heart Association (NYHA) functional class post-CRT implant, or <5% improvement (or indeed a worsening) in LVEF post CRT implant. After AF ablation, 67% of patients were free from AF at 24 months (with 46% of participants undergoing repeat ablation procedures), and there were significant improvements seen in BVP%, NYHA class and LVEF. This demonstrates AF catheter ablation is a viable, successful rhythm control strategy in the CRT population, but larger RCTs are required to demonstrate superiority over other available treatment options.

### AV node ablation in CRT

In comparison to AF ablation, there is a larger body of evidence for the use of AVNA as a treatment strategy for CRT patients with AF. In 2006, Gasparini et al. compared outcomes following CRT implantation in 3 separate groups; those with sinus rhythm (511 patients), those with permanent AF managed with medical rate control (48 patients), and those with permanent AF managed with AVNA (114 patients) (37). Those managed with AVNA had similarly significant long-term improvements in LVEF, reverse remodelling and exercise tolerance to those with sinus rhythm, whereas those with medically managed AF showed no improvement in any clinical outcomes.

**Figure 2:**
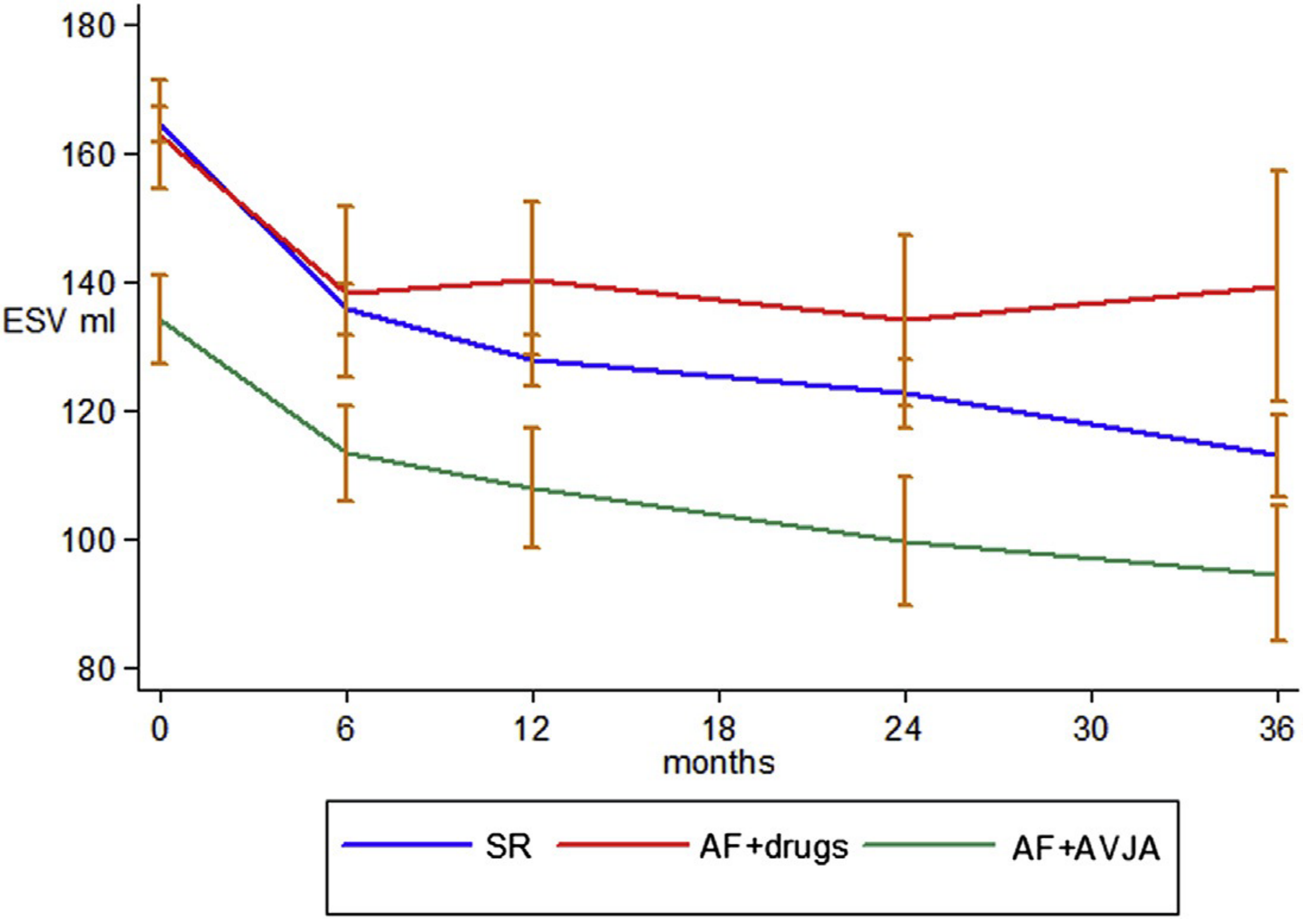
AV node ablation leads to greater LV remodelling than medical rate control over a prolonged follow-up period, with comparatively better outcomes than participants in sinus rhythm, potentially due to the higher achieved BVP. ESV, end-systolic volume (LV); SR, sinus rhythm; AVJA, AV junction ablation (equivalent to AVNA). Reproduced with permission from Gasparini et al. (38).

**Figure 3:**
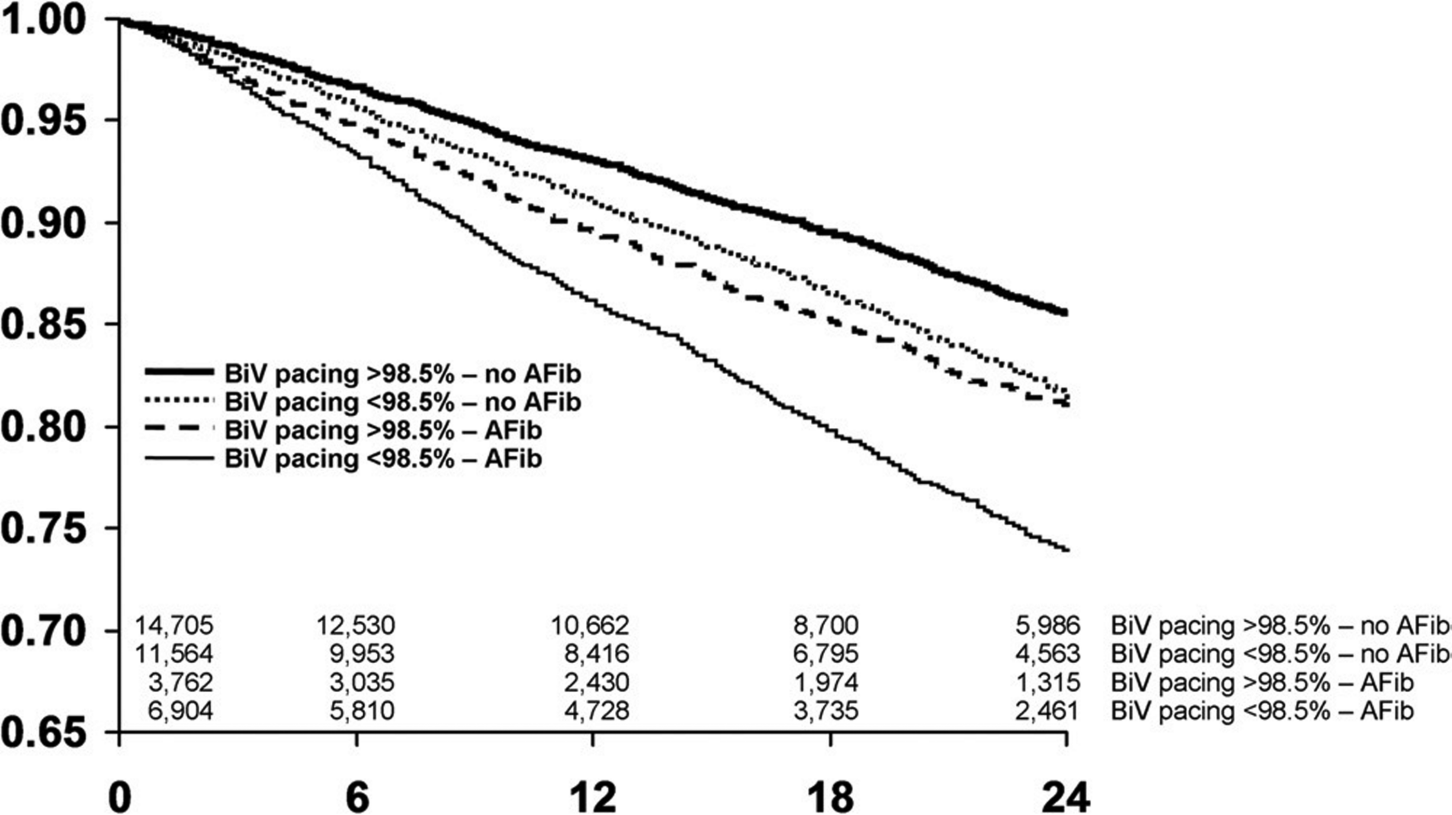
The presence of AF has an independent effect on survival separate to BVP, with worse survival in CRT patients with AF when compared to sinus rhythm patients within the same category of BVP. BVP, BiV pacing; AFib, atrial fibrillation. Reproduced with permission from Hayes et al. (7).

The CERTIFY study by the same group was a much larger prospective observational trial of over 7,000 CRT patients internationally, including 6,046 patients with sinus rhythm, 895 patients with AF on medical rate control and 443 patients with AF receiving AVNA (38). Although there were improvements in LVEF in all 3 groups, the level of improvement in the sinus rhythm and AVNA groups was significantly higher compared to the medically managed AF group. Furthermore, the sinus rhythm and AVNA groups demonstrated a sustained improvement in LV end-systolic volume (LVESV) over 3 years of follow-up, not replicated in the medically managed AF group (see Figure 2). BVP was highest in the AVNA group [96% ± 6 (mean ± SD)], followed by the sinus rhythm group (92% ± 13), and the medically managed AF group (87% ± 14). Thus, there was some correlation between BVP% and the level of effective cardiac remodelling achieved post intervention. This supports existing evidence to suggest the higher your BVP%, the lower your mortality risk following CRT implantation. Perhaps the largest evidence base for this is Hayes et al.'s cohort study of 36,935 patients followed up via remote monitoring, which demonstrated significant mortality benefit if BVP was maintained >98.5% (7). Even higher BVP levels of >99.6% conferred a further 24% reduction in mortality when compared to lower BVP%, implying ongoing mortality benefit the closer to 100% BVP one reaches. This high a BVP% is most easily achieved with AVNA vs. other AF management strategies, making AVNA an inviting option for CRT patients (38). Interestingly, however, the mere presence of AF conferred a mortality detriment when compared to sinus rhythm patients within the same BVP categories (see Figure 3); this would indicate there is an independent mortality benefit from restoring sinus rhythm in CRT patients with AF, although this does not take into account the relative inaccuracy of BVP reporting in AF (5). Thus, although AVNA largely solves the problem of low BVP, this is unlikely the sole reason for poorer CRT outcomes reported in AF patients.

A large systematic review of 31 studies with 83,571 patients by Mustafa et al. sought to evaluate the impact of AF on the effectiveness of CRT in heart failure patients, and whether having an AVNA significantly altered outcomes (16). Their results suggested that AF reduces benefit from CRT in HF with significantly higher all-cause mortality in this cohort vs. CRT patients with sinus rhythm, but AF treated with AVNA leads to similar CRT benefits experienced by those in sinus rhythm, with no significant difference in all-cause mortality between these 2 groups (OR 1.245; 95% CI: 0.914–1.696; *p* = 0.165). These results, similar to the CERTIFY study, would indicate that HF patients with AF should still be offered CRT, but with a definitive plan for its management in order to maximise BVP and overall outcomes.

Two RCTs have investigated the use of AVNA in combination with *de-novo* CRT implantation as a management strategy for symptomatic AF. The PABA-CHF trial directly compared PVI with AVNA + CRT-D implant in patients with LVEF ≤40% with ongoing paroxysmal or persistent AF despite the use of AADs (20). All patients had a narrow QRS, precluding them from conventional CRT-HF implantation, and average AF duration pre-enrolment was relatively prolonged at 4 ± 2.4 years (mean ± SD) in the PVI arm and 3.9 ± 2.8 years in the CRT-D + AVNA arm. At 6 months, there was significant improvement in LVEF in the PVI group (+8 ± 8%) when compared to the CRT-D + AVNA group (−1 ± 4%), as well as comparatively significant improvements in 6-min walk test distance (*p* < 0.001) and MLHFQ scores (*p* < 0.001). This would suggest AF ablation is preferable to AVNA + CRT-D in HF patients with drug-resistant AF, even when for relatively more prolonged AF durations.

**Figure 4:**
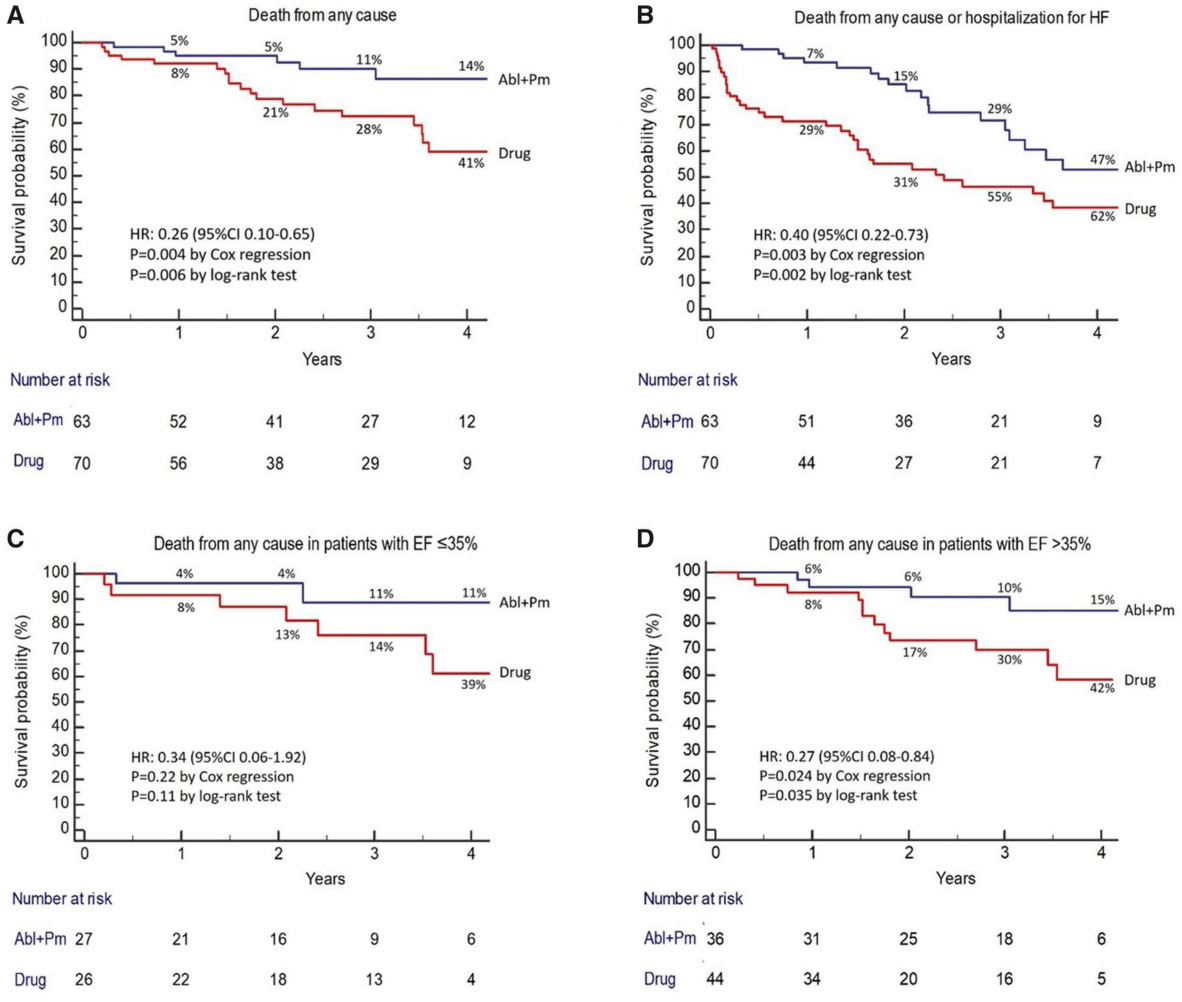
AVNA + CRT reduces the risk of death from any cause in patients with symptomatic permanent AF compared with medical therapy, in both those with EF ≤35% and >35%. Reproduced with permission from Brignole et al. (39).

Instead of PVI, the APAF-CRT mortality trial compared the effect of AVNA + *de-novo* CRT implant with OMT in a similar patient group (39). This RCT recruited 133 patients with either paroxysmal and persistent AF, all with a narrow QRS. The average duration of AF at baseline was 22 months in the AVNA + CRT arm and 19 months in the OMT arm, with all patients having either failed previous attempt(s) at AF ablation (10% of participants) or been deemed “unsuitable” for the procedure. Medical therapy was primarily in the form of rate control but included the use of AADs in 1/63 of the AVNA arm and 7/70 of the OMT arm. Over the course of a 4-year follow-up period, AVNA + CRT conferred a significant mortality benefit over a pharmacological approach [HR: 0.26 (0.10–0.65), *p* < 0.004]. This finding was reproduced in all LVEF subgroups, although did not reach statistical significance in the those with LVEF ≤35%, most likely due to a relatively small sample size (see Figure 4). In addition, 18/70 patients in the medication arm crossed over to the AVNA + CRT arm during the course of the study, mostly due to HF hospitalisation. These results seem in slight contrast to PABA-CHF, where AVNA + *de-novo* CRT offered little improvement upon baseline measures in a similar patient group. However, PABA-HF was not designed to assess long-term mortality, and with no isolated OMT arm, it is unclear how well OMT alone would have performed in comparison to AVNA + *de-novo* CRT when assessing echocardiographic and quality of life outcomes.

Although designed to review the effect of AVNA in combination with CRT, the results of PABA-CHF and APAF-CRT have limited applicability to conventional HF-CRT patients. In both trials, those receiving AVNA were compared to AF patients with a *narrow* QRS, rather than typical CRT candidates with LVEF ≤35% and QRS duration ≥120 ms. Patients receiving AVNA + CRT were also having some level of ventricular dyssynchrony *induced*, in comparison to conventional CRT which aims to *relieve* pre-existing dyssynchrony. As a result, neither PABA-HF, APAF-CRT nor CERTIFY answer the vital question of whether AVNA or AF ablation should be first line in the management of AF in patients with a pre-existing CRT implanted for conventional HF indications. An RCT directly comparing AF vs. AV node ablation in CRT patients is ongoing, which should provide clarity over how best to manage low BVP secondary to paroxysmal or persistent AF in HF patients with established CRT (40). In the meantime, as discussed thus far, we must rely on the results of individual trials of AF ablation or AVNA conducted in the CRT population, as summarised in Table 1.

**Table 1:** Summary of AF ablation and AVNA studies in the CRT population.

Study (year)	Study design	Study Duration	Treatment arms	Inclusion criteria	% with CRT at baseline	N	Baseline metrics	Median f/u (months)	Outcome(s)	Results
Gasparini et al (2006) *(AF substudy)*	Multicentre prospective observational	1995 – 2004	(A) AVNA if BVP ≤85% at 2 months* (B) OMT *Amiodarone + Digoxin discontinued at AVNA	Permanent AF CRT in situ for: • LVEF ≤35% • NYHA II-IV • QRSd ≥120ms At least 1 HFH 12 months before device implant	100%	(A) 114 (B) 48	Age: 66yrs F*: 14% LVEF: 26% QRSd: 165ms AAD**: n/a *% female **% on AADs	25	• LVEF • LVESV • Exercise capacity • CRT response (defined as reduction in LVESV ≥10% from baseline) • All-cause mortality	Improvements in AVNA vs OMT arm in: • LVEF (*p* < 0.001) • LVESV (*p* < 0.001) • Exercise capacity (*p* < 0.001) • CRT response (68% vs 18%; *p* = 0.001) No significant improvement in same parameters in OMT arm from baseline Higher mortality in OMT arm (OR 11.1, *p* < 0.001)
Gasparini et al (2008) *(AF substudy)*	Multicentre prospective observational	1995 – 2004	(A) AVNA if BVP ≤85% at 2 months* (B) OMT *Amiodarone + Digoxin discontinued at AVNA	Permanent AF CRT in situ (for any reason)	100%	(A) 118 (B) 125	Age: 66yrs F: 22% LVEF: 26% QRSd: 162ms AAD: 54%	34	All-cause mortality	Lower mortality in AVNA vs OMT arm (HR 0.31, *p* = 0.048)
PABA-CHF (2008)	Multicentre RCT	2002 – 2006	(A) AF ablation (B) AVNA + de-novo CRTD	pAF or persistent AF despite AADs NYHA II or III LVEF ≤ 40%	n/a	(A) 41 (B) 40	Age: 61yrs F: 9% LVEF: 28% **QRSd: 91ms** AAD: 100%	n/a *(100% present at 6mo f/u)*	• LVEF • 6MWD • MLHFQ • Freedom from AF	Greater improvements in AF ablation vs AVNA+CRTD arm in: • LVEF (+8% vs +1%, *p* < 0.001) • 6MWD (+71m vs +16m, *p* <0.001) • MLHFQ (-29 vs -7, *p* < 0.001) • Freedom from AF (88% vs 0%)
Dong et al (2010)	Single centre prospective observational	2002 – 2006	(A) AVNA (B) OMT	CRT in situ for: • LVEF ≤35% • NYHA II-IV despite OMT • QRSd ≥120ms	100%	(A) 45 (B) 109	Age: 70yrs F: 15% LVEF: 25% QRSd: 168ms AAD: 19%	25	• NYHA • LVEF • LVEDD • All-cause mortality	Greater improvements in AVNA vs OMT arm in: • NYHA class (*p* = 0.004) • Survival at 2 years (HR 0.13, *p* = 0.007) Comparable improvements in both AVNA and OMT arms in: • LVEF (8.1% vs 6.8% *p* = 0.49) • LVEDD (-2.1 mm vs -2.1 mm, *p* = 0.74)
CERTIFY (2013) *(AF substudy)*	Multicentre prospective observational	1999 – 2011	(A) AVNA (B) OMT	Permanent AF CRT in situ for: • LVEF ≤35% • NYHA II-IV • QRSd ≥ 120ms	100%	(A) 443 (B) 895	Age: 69yrs F: 16% LVEF: 27% QRSd: 157ms AAD: 29%	37	• All-cause mortality • Cardiac mortality • LVEF	Lower all-cause mortality (HR 0.67, *p* < 0.001) and cardiac mortality (HR 0.63, *p* < 0.003) in AVNA vs OMT arm Greater improvement in AVNA vs OMT arm in LVEF at 6 months (+8% vs +4%; *p* < 0.001)
ARC-HF (2013)	Single centre RCT	2009 – 2012	(A) AF ablation (B) Medical rate control	Persistent AF (> 7 days) NYHA II-IV LVEF ≤35%	(A) 31% (B) 12%	(A) 26 (B) 26	Age: 63yrs F: 23% LVEF: 24% QRSd: 116ms (non-paced only) AAD: 12%	n/a (98% present at 12mo f/u)	• Peak VO2 • MLHFQ • LVEF • 6MWD • BNP	Improvement in ablation arm vs minor reduction in drug arm in peak VO_2_ (+3.07ml/kg/min difference between arms, *p* = 0.018) Greater improvement in ablation vs drug arm in: • MLHFQ (-21 vs -8, *p* = 0.019) • BNP (-124ng/L vs -18ng/L, *p* = 0.045) Greater, but nonsignificant improvement in ablation vs drug arm in LVEF (+10.9% vs +5.4%, *p* = 0.055) No significant difference in 6MWD
AATAC-AF (2016)	Multicentre RCT	Not specified	(A) AF ablation (B) AAD (Amiodarone)	Persistent AF ICD or CRTD in situ NYHA II or III LVEF ≤40%	100% with either ICD or CRTD (% with CRT not specified)	(A) 102 (B) 101	Age: 61yrs F: 26% LVEF: 30% QRSd: n/a AAD: (A) 0% (B) 100%	No patients lost to f/u at end of 24 month study period *(13% mortality overall)*	• All-cause mortality • Unplanned hospitalisations • LVEF • MLHFQ • 6MWD • AF burden	Greater improvement in ablation vs AAD arm in: • LVEF (+8.1% vs +6.2%, *p* = 0.02) • MLHFQ (-14 vs -2.9, *p* <0.001) • 6MWD (+27m vs 8m, *p* <0.001) AF more likely to recur in AAD vs ablation arm (HR 2.5, *p* <0.001) Lower mortality in ablation vs AAD arm (8% vs 18%, *p* = 0.037) Lower unplanned hospitalisation rate in ablation vs AAD arm (31% vs 57%, *p* <0.001)
Gasparini et al. (2018)	Multicentre prospective observational *(pooled analysis from 2 RCTs and 1 observational trial)*	2004 – 2014	(A) AVNA if BVP ≤95% at 3 months (B) OMT	Permanent AF CRT in situ for: • LVEF ≤35% • NYHA II-IV • QRSd ≥120ms	100%	(A) 262 (B) 402	Age: 69yrs F: 15% LVEF: 27% QRSd: 142ms AAD: 21%	18	• ICD shocks (appropriate and inappropriate) • All-cause hospitalisations	Large reductions in AVNA arm vs OMT arm in: • Appropriate shocks (IRR 0.23, *p* <0.001) • Inappropriate shocks (IRR 0.09, *p* <0.001) Lower all-cause hospitalisations in AVNA arm (IRR 0.57, *p* < 0.001)
CASTLE-AF (2018)	Multicentre RCT	2008 – 2016	(A) AF ablation (B) OMT	pAF or persistent AF NYHA II-IV LVEF ≤35% ICD or CRTD Either no response to, unacceptable SEs from or unwilling to take AADs	28%	(A) 179 (B) 184	Age: 64yrs F: 15% LVEF: 32% QRSd: n/a AAD: 59% *(with either no response to AADs or intolerable SEs)*	38	• All-cause mortality • Cardiac mortality • HFH	Lower chance in ablation vs OMT arm of: • All-cause mortality (HR 0.53, *p* = 0.009) • Cardiac mortality (HR 0.49, *p* = 0.008) • HFH (HR 0.56, *p* = 0.004)
AMICA trial (2019)	Multicentre RCT	2008 – 2017	(A) AF ablation (B) OMT +/-DCCV	Symptomatic or longstanding (1-4 yrs) persistent AF NYHA II or III Indicated for ICD or CRTD LVEF ≤35% LAd <60mm	24% at enrolment 44% at discharge	(A) 68 (B) 72	Age: 65yrs F: 10% LVEF: 27% QRSd: n/a AAD: 32%	12	• All-cause mortality • SAEs • LVEF • MLHFQ • 6MWD • NT-pro BNP • AF burden	Comparable improvements in ablation and OMT arms in: • LVEF (+8.8% vs +7.3%, *p* = 0.36) • MLHFQ (-11.2 vs -8.9, *p* = 0.42) • 6MWD (+46m vs +81 m, *p* = 0.07) • NT-proBNP (-891ng/L vs - 419ng/L, *p* = 0.60) No differences between arms in mortality (8.2% vs 8.0%) or proportion of patients with >1 SAE (65% vs 56%, *p* = 0.19)
Fink et al. (2019)	Single centre retrospective observational	2010 – 2017	AF ablation	CRT nonresponders, defined as ≥1 of: • BVP <95% due to AF • <1 point ΔNYHA • ≤5% ΔLVEF	100%	38	Age: 68yrs F: 21% LVEF: 30% QRSd: n/a AAD: 71%	27	• Freedom from AF • BVP • LVEF • NYHA	67% freedom from AF at 2 yrs Improvements from baseline in: • BVP (+8% *p* < 0.001) • LVEF (+2.2% *p* = 0.023) • NYHA class (*p* < 0.0001)
RAFT-AF (2022)	Multicentre RCT	2011 – 2018	(A) AF ablation (B) OMT +/-AVNA if needed *(% undergoing AVNA not specified)*	NYHA II or III despite OMT Elevated BNP High burden pAF or persistent AF <3 yrs LAd <55mm	13%	(A) 214 (B)197	Age: 67yrs F: 26% LVEF: 40% QRSd: n/a AAD: 42%	37	• All-cause mortality • HF event • MLHFQ • 6MWD • NT-pro BNP • LVEF	Non-significant trends in ablation vs OMT arm towards lower: • All-cause mortality (HR 0.71, *p* = 0.066) • HF events (HR 0.79, *p* = 0.349) Greater improvements in ablation vs OMT arm at 2 yrs in: • MLHFQ (*p* = 0.0036) • 6MWD (*p* = 0.025) • NT-proBNP (*p* < 0.0001) • LVEF (+10.1% vs + 3.8%, *p* = 0.017)
APAF-CRT (2021)	Multicentre RCT	2014 – 2020	(A) AVNA + de-novo CRT (B) OMT	Severely symptomatic permanent AF where AF ablation had failed or deemed unsuitable QRS ≤110ms ≥1 HFH in last yr	n/a	(A) 63 (B) 70	Age: 73yrs F: 36% LVEF: 41% QRSd: 95ms AAD: 6%	29	• All-cause mortality • HFH	Lower all-cause mortality in AVNA+CRT vs OMT arm (HR 0.26, *p* = 0.004), but • only significant when stratified for LVEF >35% (HR 0.27, *p* = 0.02) • nonsignificant in pts with LVEF ≤35% (HR 0.34, *p* = 0.22) Lower rate of combined endpoint of all-cause mortality or HFH in AVNA+CRT arm (HR 0.40; *p* = 0.002)

6MWD = 6 minute walk distance; AAD = anti arrhythmic drug; AF = atrial fibrillation; AVNA = atrioventricular node ablation; BNP = B type natriuretic peptide; BVP = biventricular pacing; CRT = cardiac resynchronisation therapy; CRTD = cardiac resynchronisation therapy with defibrillator; DCCV = direct current cardioversion; f/u = follow up; HF = heart failure; HFH = heart failure hospitalisation; HR = hazard ratio; ICD = implantable cardioverter defibrillator; IRR = incidence rate ratio; LAd = left atrial diameter; LVEDD = left ventricular end diastolic diameter; LVEF = left ventricular ejection fraction; LVESV = left ventricular end systolic volume; MLHFQ = Minnesota living with heart failure questionnaire; NT-proBNP = N-terminal pro B type natriuretic peptide; NYHA = New York Heart Association; OMT = optimal medical therapy; OR = odds ratio; pAF = paroxysmal AF; QRSd = QRS duration; RCT = randomised controlled trial; SAEs = serious adverse events; SEs = side effects; VO_2_ = peak oxygen consumption.

### The role of AV synchrony in CRT

AVNA provides the greatest chance of achieving inter-ventricular (VV) synchrony in AF-CRT patients; (38) however, unlike AF ablation, it does not address the issue of *AV* dyssynchrony. In the absence of data directly comparing AVNA to AF ablation in CRT, it is unclear how important restoring AV synchrony is for CRT patients with AF, and thus how much extra benefit AF ablation may provide over AVNA.

In sinus rhythm patients, the level of intrinsic AV delay (AVD), represented by PR interval at baseline, has been shown to predict outcomes from CRT. In a subgroup analysis of the MADIT-CRT trial, Kutyifa et al. demonstrated a more than two-fold increased risk of death in non-LBBB patients with a PR interval <230 ms receiving CRT-D compared to ICD (HR: 2.14; 95% CI: 1.12–4.09; *p* = 0.022), whereas those with a PR interval ≥230 ms experienced a 81% decrease in risk of all-cause mortality from CRT-D compared to ICD therapy (HR: 0.19; 95% CI: 0.13–0.57; *p* < 0.0001) (41). Similarly, Olshansky et al.'s subgroup analysis of the COMPANION trial, which randomised HFrEF patients to either CRT-P, CRT-D or OMT, found those with prolonged PR intervals ≥200 ms had a greater reduction in risk of all-cause mortality or heart failure hospitalisation from CRT vs. OMT (HR: 0.54; *p* < 0.01) when compared to those with normal PR intervals (HR: 0.71; *p* = 0.02) (42). These analyses would indicate CRT's mortality benefit is partially incurred by its role in correcting intrinsic AV dyssynchrony, providing relatively less benefit to those without this at baseline. This mirrors similarly poorer outcomes from CRT in those without sufficient baseline VV dyssynchrony (43, 44), and provides support for the restoration of AV synchrony via curative catheter ablation in CRT patients with AF.

**Figure 5:**
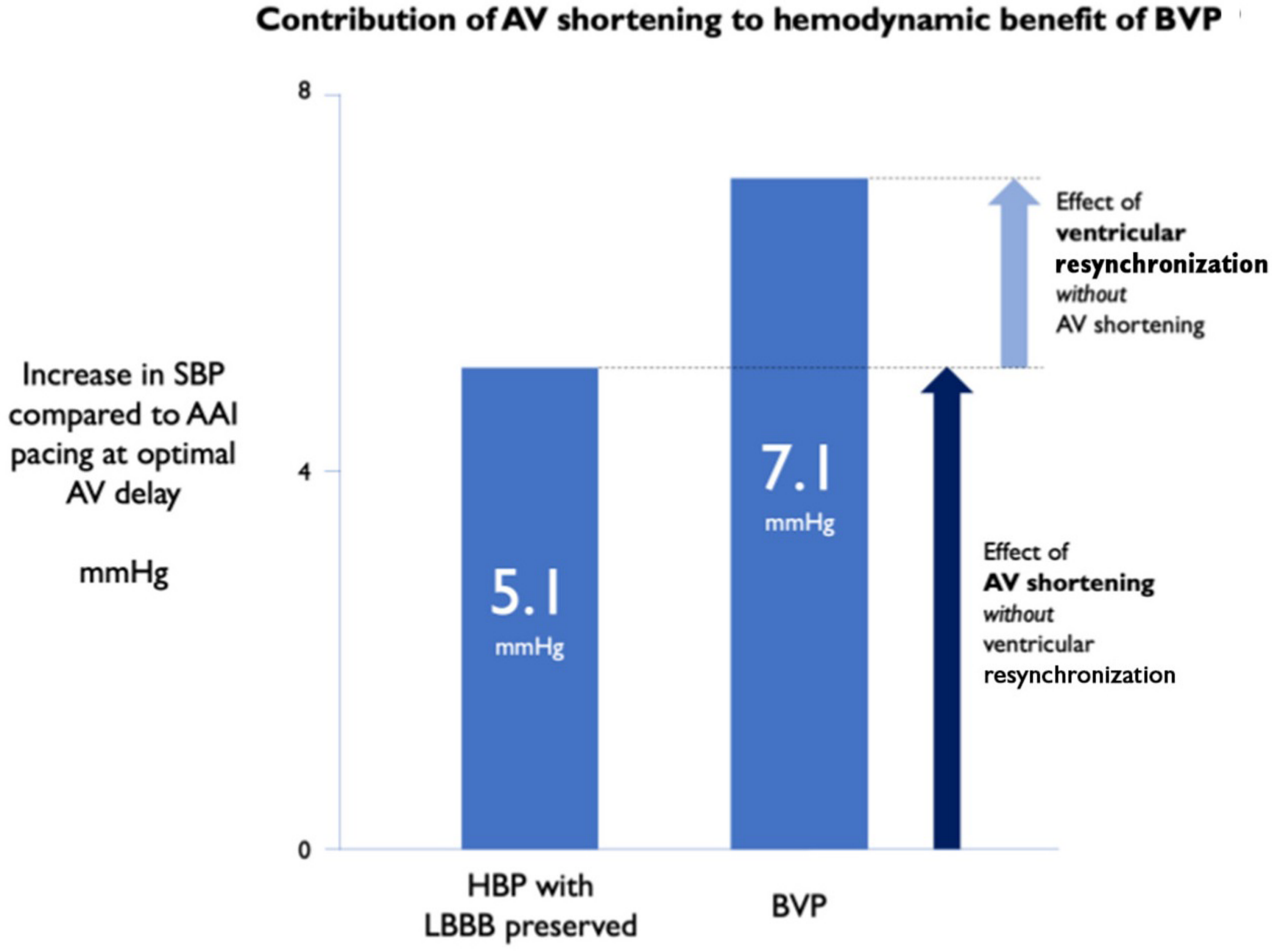
AV shortening without ventricular resynchronisation provides the majority of acute haemodynamic benefit from CRT. SBP, systolic blood pressure; HBP, His-bundle pacing. Reproduced with permission from Arnold et al. (46).

At a physiological level, observational studies have shown optimising AVD in CRT patients has an acutely positive haemodynamic impact (45). In fact, in one recent temporary pacing study of LBBB patients referred for conventional CRT, shortening of the AVD was shown to provide the *majority* of acute haemodynamic benefit derived from temporary BVP, more so than any effect of VV resynchronisation (see Figure 5) (46). These initial data are certainly a signal towards AV synchrony being of significant physiological importance in CRT.

Despite these findings, AVD optimisation strategies have failed to reliably demonstrate clinical benefit among the general CRT population. In the SMART-AV trial, 1,014 CRT-D patients were randomised to receive either echocardiographic AVD optimisation, optimisation with SmartDelay (an electrogram-based algorithm), or a fixed AVD of 120 ms (47). At 6-month follow up, AVD optimisation with either method failed to derive any significant improvement in LV remodelling or quality of life over the implementation of an empirical AVD, indicating no added benefit from optimising AV synchrony by these methods. However, a fixed AVD of 120 ms was already providing some level of benefit in these patients, including a median reduction in LVESV by −15 ml (range −45 ml to +6 ml) and median improvement in LVEF by +5.1% (range −1.0% to +13.1%), which likely limited the degree of additional benefit further AVD optimisation could offer. Indeed, in the BRAVO trial, which randomised CRT patients to either echocardiographic or haemodynamic methods of calculating optimal AVD, 70% of patients’ optimal AVD was within 20 ms of 120 ms, with results indicating small differences in AVD near the optimum have relatively little impact on CRT outcomes (48). However, for 30% of patients, their optimal AVD was >20 ms beyond the standard 120 ms delay, indicating there is a subset of patients where precise CRT optimisation is of benefit. This hypothesis is supported by a small study of 39 CRT non-responders (defined as ≤5% improvement in LVEF), or incomplete responders (final LVEF ≤40% at least 3 months post-CRT), where CRT optimisation using electrical dyssynchrony mapping (EDM) significantly improved LVEF [+4.5% ± 5.9 (mean ± SD), *p* < 0.001] and LVESV (−10.5 ml ± 23.8, *p* = 0.009) at 6-month follow-up (49). Their optimisation method resulted in 10 of the 39 participants having their AVD altered, with baseline AVDs deemed optimal in the remainder during individual EDM generation.

Altogether, manipulation of AVD appears to be integral to the effective delivery of CRT in a significant proportion of sinus rhythm patients. This would suggest that restoring and optimising AV synchrony via AF ablation may convey better outcomes in AF patients with CRT compared to AVNA, which by contrast renders AV synchrony obsolete. Restoring sinus rhythm also reinstates mechanical atrial systole, which in some reports contributes up to 30% of total cardiac output (50). As mentioned previously, an RCT directly comparing AF vs. AV node ablation should provide clarity over the exact importance of AV synchrony and atrial systole in the CRT population (40).

### When to stop chasing sinus rhythm

Despite its apparent importance in the effective delivery of CRT, the restoration of sinus rhythm must be weighed up with the relative disadvantages of AF ablation in this cohort, including a significantly higher procedural risk profile, and relatively high recurrence rates in the HF population; one aforementioned registry reported an AF recurrence rate of up to 52% in HF patients (32). Recurrence comes with cumulative procedural risk and a significant financial impact on health services (51). Repeat procedures and complications also have a significant psychological impact on the individual, who may have already suffered traumatic medical events in the lead up to receiving CRT.

Patient selection is therefore key when considering which CRT patients to refer for AF catheter ablation over AVNA, aiming to select those with the lowest chance of recurrence. As well as prioritising those with early-onset, paroxysmal AF over long-standing persistent forms, features such as LA volume (3, 52) and fibrosis burden (3) can also be helpful in stratifying the likelihood of success from curative AF ablation in CRT patients.

With regards to what exact cut-off to use for “long-standing” AF, definitions in clinical trials have varied widely between 6 months and 3 years (8, 21, 53), further complicated by the unknown duration of preceding asymptomatic arrhythmia in those without devices. There is also discrepancy between whether to utilise *pre-enrolment* or *pre-intervention* AF duration, a vital distinction to make when international waiting times for AF ablations continue to climb. Qeska et al.'s observational study of 6,253 patients (including 18.8% with unspecified HF) referred for first AF ablation suggests long wait-times are associated with substantial morbidity risk; however, by not adjusting for initial AF diagnosis-to-referral time [which averaged 741 ± 581 days (mean ± SD) across the cohort], it is difficult to accurately determine the proportional impact long wait-times had compared to any initial delay in referral (54). In contrast, Kalman et al.'s recently published RCT suggests delaying first AF ablation from 1 month to 12 months after enrolment makes no difference to ablation success rates at 1 year in patients with either paroxysmal or persistent AF (HR: 1.12; 95% CI: 0.59–2.13, *p* = 0.7). However, all participants had to have an AF duration of less than 1 year prior to enrolment, and it is unclear what methods were used to ascertain pre-enrolment AF onset. The exact split of methods used for post-ablation AF burden assessment (which could be either via a pre-existing implantable loop recorder or pacemaker, twice-daily mobile ECG monitoring or intermittent 24 h holter monitoring) was also unreported between groups, potentially skewing any final results (55).

Overall, it is highly likely AF ablation is futile beyond a certain duration of arrhythmia, but an exact cut-off point has yet to have been convincingly identified. CRT patients with new-onset AF are in a unique position to provide robust data on the exact impact of arrhythmia duration on rhythm control success rates, as both symptomatic and subclinical AF can be detected with immediacy. Future trials comparing the success rates of AF ablation in CRT patients with differing AF duration should elucidate a realistic deadline for considering AF ablation over AVNA, minimising any risk of recurrence with its associated negative sequalae.

## Other supraventricular arrhythmias

AF is not the only form of SVT to commonly arise in CRT patients, with one large multi-centre study detecting episodes of unifocal AT in 21% of new CRT implants (56). Much like AF, non-AF SVTs such as AT, atrioventricular nodal re-entrant tachycardia (AVNRT) and atrial flutter can interfere with effective BVP delivery. They also account for a large proportion of inappropriate shocks from implantable cardioverter-defibrillators (ICDs), which many CRT patients have concurrently implanted in the form of CRT-D (57, 58). Not only are inappropriate shocks psychologically traumatic, but they have also been independently associated with increased long-term mortality in ICD patients (57). Minimising interference from SVTs in CRT-D patients is therefore not only important for allowing effective BVP, but also to prevent the detrimental short-term and long-term impacts of inappropriate shock therapy.

In one retrospective study of ICD and CRT-D patients between 2005 and 2009, non-AF SVTs were monitored and subsequently managed with ablation. Non-AF SVTs occurred in 11.8% of CRT-D patients and 13.4% of ICD patients during the course of the 5-year study period (59). Despite the presence of SVT discriminator technology on every device, 26 of the 84 patients enrolled received inappropriate ICD shocks for non-AF SVT, with an average of 4.2 shocks per patient in this group. 22 patients went on to receive radiofrequency ablation for their arrhythmia, with 93% of SVTs successfully ablated. During the subsequent 20.7 ± 11.9 month follow-up period, 95% of these successfully ablated patients remained free of ICD shock therapies, whereas 62.5% of those with non-inducible SVTs, or unsuccessfully ablated SVTs, had further shocks during the same follow-up period. It is important to note that this study predates the MADIT-RIT trial, which provided important updates to ICD and CRT-D programming; thus it is likely these levels of inappropriate shock therapy would now be significantly lower with modern device programming methods (60, 61).

Unlike AF, ablation of non-AF SVTs confers much lower rates of recurrence of the original arrhythmia, with one large cohort study of 2,260 patients reporting arrhythmia recurrence after AVNRT, AVRT or AT ablations in only 3.5% patients over a median follow-up period of 1,856 (range 1,125–2,677) days (62). Given these low recurrence rates, the threshold for considering catheter ablation for non-AF SVTs is comparatively lower than for its AF counterpart. This is reflected in the European Society of Cardiology (ESC) guidelines which advocate catheter ablation as first-line therapy for patients with inappropriate shock therapy secondary to recurrent SVT (63, 64). There is no reason not to also strongly consider catheter ablation in the management CRT patients with ineffective BVP secondary to non-AF SVT despite optimal medical rate control.

## Ventricular arrhythmias

By virtue, patients referred for CRT are significantly more likely than the average population to experience VAs. Class I & II indications for CRT are primarily based on the presence LVEF ≤35% and a broad QRS on ECG, both of which are associated with an increased risk of VAs (65, 66).

CRT itself has been shown to reduce the incidence of appropriate ICD therapy for VAs (67), most significantly in those with the greatest LVEF recovery post implant (11). There is also some indication that the recent progress made in medical therapy for HF, most notably with neprilysin inhibitors and SGLT2 inhibitors, has contributed to a relative decline in the levels of sudden death in HFrEF patients (68). Despite this, VAs such as NSVT and PVCs remain significant causes of low BVP and subsequent CRT non-response (1, 2), with significant associations with mortality in CRT patients (69). We will review the current evidence regarding medical and procedural management of VAs in this cohort.

### Medical management of VAs in CRT

First-line treatment of VAs in CRT patients involves optimising the management of any underlying cardiac disease which increases VA risk, such as undertaking coronary angioplasty or optimising HF therapy. The majority of CRT patients will already be on beta-blocker medication to reduce intrinsic conduction, and this should be uptitrated to the maximum tolerated dose to suppress VAs. Additional AADs may then be required if there is significant VA burden despite maximal beta-blocker therapy.

The most commonly used AAD is amiodarone. Multiple trials over the years have demonstrated amiodarone's effectiveness in reducing VA burden in HF patients. For example, in the international multicentre Optimal Pharmacological Therapy in Cardioverter Defibrillator Patients (OPTIC) study, the addition of amiodarone significantly reduced the risk of shocks for VAs in ICD patients with LVEF <40% when compared to those on beta-blocker therapy alone, with an impressive a hazard ratio of 0.27 (95% CI: 0.14–0.52; *p* < 0.001) (70).

Although amiodarone is clearly effective at suppressing VAs, this does not translate into a mortality benefit. In the Sudden Cardiac Death in Heart Failure Trial (SCD-HeFT), amiodarone had no impact on survival in HF patients with LVEF ≤35%, compared to ICD therapy, which by contrast was associated with a 23% reduction in overall mortality (71). This is most likely due to amiodarone's unfavourable safety profile, with potential side effects including thyrotoxicosis, hepatotoxicity and pulmonary fibrosis, all associated with significant morbidity and mortality. This has been demonstrated in the CRT population; Adelstein et al. compared 37 patients already established on amiodarone therapy to 30 amiodarone-naïve patients receiving upgrades to CRT-D from secondary-prevention ICDs (72). Not only did amiodarone confer a significantly increased risk of their composite endpoint of all-cause mortality, heart transplant or LV-assist device (LVAD) implant (HR: 2.26; 95% CI: 1.20–4.23; *p* = 0.011), but adverse effects were experienced by 5/37 (14%) patients taking amiodarone during the median 29-month follow-up period, including 2 cases of possible lung toxicity, and 1 case of hyperthyroidism with subsequent thyroidectomy. The use of amiodarone was also associated with a significant reduction in QRS narrowing (8 vs. 20 ms; *p* = 0.021) and less LVEF improvement post-CRT (−2.7% vs. +5.2%; *p* = 0.006), indicating that not only does amiodarone impact mortality in CRT patients, but it also directly impairs CRT efficacy.

Similar findings came from a larger observational analysis of the German DEVICE registry (73). The initial aim of the study was to investigate the impact of amiodarone on defibrillation threshold (DFT) testing when prescribed in addition to beta-blocker therapy. To the authors’ surprise, they demonstrated a more than two-fold increase in 1-year all-cause mortality in patients receiving amiodarone in addition to beta-blockers compared to those on beta-blockers alone (adjusted HR: 2.09, *p* < 0.001). The majority of patients had LVEF ≤30% (2,771/4,338 patients), with similar numbers in each medication group receiving CRT in addition to ICD implants (29.4% of beta-blocker alone group vs. 32% of amiodarone + beta-blocker group). To reconcile the confounding impact of VAs on mortality, an additional subgroup analysis was conducted comparing patients with primary and secondary ICD indications; within this, amiodarone still conferred a significantly higher all-cause mortality, suggesting its impact on mortality is independent of VA burden.

Overall, this suggests amiodarone is best used as a potent suppressor of acute VAs in short-term settings, with compelling evidence against its long-term use in the CRT population.

Beyond amiodarone, the AAD options for CRT patients are limited due to many being contraindicated in severe HF. This is mostly due to side effects of negative inotropy (such is the case for mexiletine) (74), or associations with excess mortality (75), most infamously published in the CAST trial with regard to class 1c AADs (76). These contraindications have their own controversies, beyond the scope of this review article.

We do, however, have some evidence for the use of sotalol as an AAD in the HF population. In 1999, Pacifico et al. demonstrated a 51% reduction in the risk of any shock or death in ICD patients with LVEF ≤30% taking sotalol vs. placebo (77). However, this level of risk reduction has not been reliably replicated since; in a systematic review of 5 RCTs comparing sotalol to control medical therapy [including Pacifico et al. (77)], sotalol did not significantly reduce VA episodes [odds ratio (OR): 0.83; 95% CI: 0.42–1.65; *p* = 0.594] (78). Although the majority of patients included in the analysis had LVEF ≤40%, many predated CRT, and all occurred before the most recent advances in HF medical therapy. It is therefore possible sotalol confers even *less* anti-arrhythmic benefit over OMT in the modern HF era. Overall, it is clear sotalol offers less anti-arrhythmic potency than amiodarone, but with a relatively safer risk profile, it may be considered as an AAD for CRT patients with VAs where amiodarone is not suitable, or longer term AAD therapy is required.

### Catheter ablation for ventricular arrhythmias

Catheter ablation is another option for CRT patients on maximal tolerated medical therapy with ongoing low BVP secondary to VAs. In a prospective observational study by Lakkireddy et al. the effect of PVC ablation was assessed in 65 CRT non-responders with a high burden of PVC on holter monitoring (>10,000 PVCs over 24 h) (79). Mean BVP pre-ablation was 76% (range 37–90), which improved to a mean of 98% (range 96–100) post procedure (*p* < 0.001) at 1-year follow up. 88% of patients maintained a low PVC burden of <1,000/day at 1 year, with only 1 patient undergoing repeat ablation in the follow-up period. Echocardiography at 6 months post-ablation demonstrated significant improvement in LVEF and LV dimensions, with an average improvement in LVEF from 26.2% ± 5.5 to 32.7% ± 6.7 (mean ± SD) (*p* < 0.001). Those with the highest pre-ablation PVC burden of >22% had the greatest improvement in LVEF after ablation, whereas of the 34% of patients who remained CRT non-responders post-ablation, (defined as <5% improvement in LVEF), PVC burden pre-ablation had been significantly lower at 16.2 ± 5.0%. Interestingly, these patients’ LVEF did not improve *despite* a significant improvement in BVP from 83.8% to 99.8% and a similarly high ablation success rate (88.9%) to their CRT responder counterparts (91.8%). This reiterates the point that although is it extremely important to maintain BVP >95% (1), there are other causes of CRT non-response independent of low BVP%.

**Figure 6:**
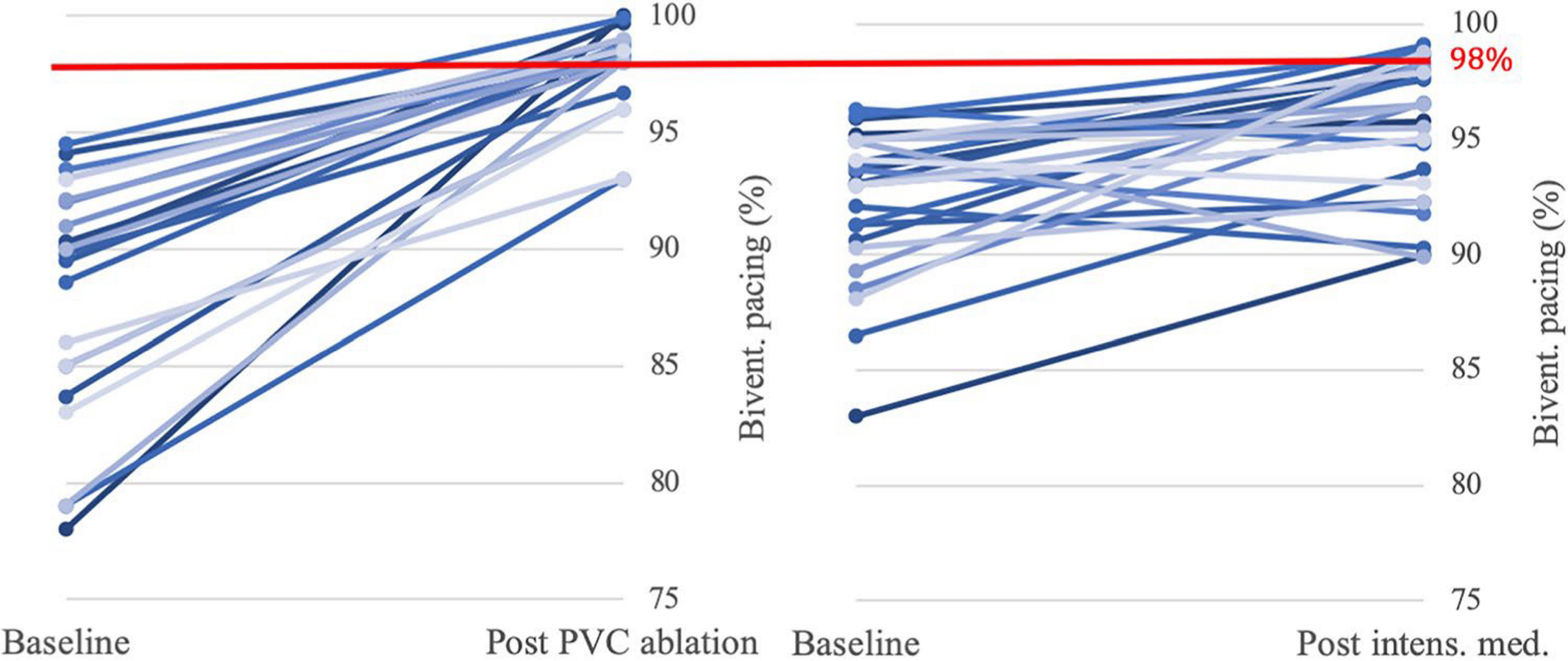
Impact of PVC ablation (22 patients) and intensified medical therapy (27 patients) on BVP. Significantly more patients in the PVC ablation group (16/22, 73%) reached the target BVP of >98% than in the medical therapy group (6/27, 22%) (*p* < 0.001). Reproduced with permission from van den Bruck et al. (80).

A more recent analysis by van den Bruck et al. retrospectively compared outcomes between CRT patients with low BVP (mean BVP 88.1% ± 10.9%) who underwent either PVC ablation (22/64), VT ablation (15/64) or intensified medical treatment (27/64) (80). Intensification of medical therapy involved uptitration of beta-blockers, and the addition of oral amiodarone in 4 of the 27 patients in this group. Despite having a lower baseline BVP, a significantly greater proportion of those in the ablation groups reached a BVP ≥98% after treatment when compared to the medical therapy group (*p* < 0.001). The greatest improvement in BVP was amongst the VT ablation group, with a mean BVP improvement of +16.3% (range 3.3–32.7) vs. +9.9% (range 1.2–47.6) in the PVC ablation group and only +3.2% (range −5.0 to 10.7) in the medical therapy group (see Figure 6). These improvements in BVP were associated with a greater improvement in functional status, with 54% of patients receiving ablation improving from NYHA class III to II vs. only 7% of the medically managed patients (*p* = 0.003). Thus, although optimising medical therapy modestly improved patient outcomes, catheter ablation demonstrated significant additional benefit in comparison. RCT-level evidence comparing the efficacy of medical therapy vs. catheter ablation in the management of low BVP secondary to VAs is lacking, but the above data would suggest ablation is a viable, effective option for these patients.

Of note, the majority of VAs ablated in these trials were from a single myocardial focus. Ablation is comparatively more challenging in those with *multifocal* PVCs, which accounts for approximately 1 in 5 patients referred for PVC ablation (81). Multifocal PVC ablation has, however been shown to be viable treatment option with the latest morphology-matching software, with a reported five-fold reduction in PVC burden in one recent European trial (81).

In CRT patients with an ICD, results from the PARTITA trial would also suggest considering early VT ablation after the first appropriate ICD shock, with this approach reducing risk of death or HF hospitalisation when compared to controls who did not receive ablation after their first ICD shock (82). Appropriate shocks were also predicted by the preceding occurrence of anti-tachycardia pacing (ATP), which makes this a valuable warning sign for impending shock therapy. Future trials may determine whether there is critical level of ATP that warrants ablation for the prevention of future shock therapy in defibrillator patients.

## The role of non-AAD medication in CRT arrhythmia management

CRT allows for the uptitration of HF therapy via its augmentation of blood pressure and prevention of bradycardia (83). However, HF medication has its own reciprocal impact to be considered when addressing its role in the management of arrhythmia in CRT.

### Are SGLT2 inhibitors anti-arrhythmic?

The recent advent of novel drug therapies has provided much excitement in the HF community. Sacubitril/valsartan was the first drug in over a decade to demonstrate a mortality benefit in HF (84). However, despite significantly reducing the risk of sudden cardiac death, it has not been shown to significantly impact arrhythmia burden (85).

By contrast, SGLT2 inhibitors have produced exciting results related to their potential anti-arrhythmic properties. In diabetic patients prescribed SGLT2 inhibitors instead of other diabetic medication in addition to metformin, Duran et al. (86) observed beneficial alterations in ventricular repolarisation dispersion, which in turn is associated with the development of cardiac arrhythmias (87). SGLT2 inhibitors also interfere with redox signalling in cardiomyocytes, disruption of which can lead to atrial arrhythmias (88).

These anti-arrhythmic properties have translated into real-world outcomes. A *post hoc* analysis of the DECLARE-TIMI-58 trial demonstrated a reduced incidence of AF episodes in patients receiving Dapagliflozin, including in those with “high risk” features of diabetes, atherosclerotic cardiovascular disease or HF, and in those with a previous history of AF (89). Similarly, a *post hoc* analysis of the DAPA-HF trial demonstrated a significant reduction in the composite endpoint of serious ventricular arrhythmia, cardiac arrest, or sudden death in patients receiving Dapagliflozin in addition to their standard HF therapy (HR: 0.79; 95% CI: 0.63–0.99; *p* = 0.037) (90). A similar, albeit not statistically significant reduction was seen in “serious” ventricular arrhythmia, with a HR: 0.76 (95% CI: 0.53–1.10).

It is possible that these anti-arrhythmic properties of SGLT2 inhibitors contributed to the reduction in cardiovascular mortality seen in the landmark DAPA-HF (91), EMPEROR-Reduced (92) and EMPA-REG OUTCOME (93) clinical trials. One trial investigating the direct impact of SGLT2 inhibitors on arrhythmia burden in CRT patients is ongoing, but does not include BVP efficacy or cardiac remodelling as endpoints (94). Regardless, we would recommend the use of SGLT2 inhibitors in CRT patients for both HF optimisation and anti-arrhythmic purposes, which may well prove to beneficially impact BVP% and reduce the risk of appropriate and inappropriate shocks in those with CRT-D.

### Digoxin—friend or foe?

Another commonly used medication in CRT patients is digoxin. However, its role in the treatment of HF is currently the topic of ongoing debate due to reported associations with mortality and ventricular arrhythmia. In one sub-analysis of the landmark MADIT-CRT trial, which randomised 1,820 patients with HFrEF to receive either CRT-D or ICD implants, participants taking digoxin had a significantly higher cumulative risk of high-rate VAs compared to those not taking digoxin (22% and 15% respectively; *p* = 0.005), with the use of digoxin independently increasing risk of VAs by 41% (*p* = 0.002) and risk of “high-rate VAs” (≥200 beats/min) by 65% (*p* ≤ 0.001) (95). Although digoxin use was not significantly associated with mortality (*p* = 0.096), the authors note that all the MADIT-CRT participants had defibrillators implanted, providing independent significant mortality reduction. With a significantly increased risk of VAs, digoxin users may well have experienced increased risk of death had this not been mitigated by the implantation of CRT-D and ICD devices.

The largest RCT of digoxin use to date is the DIG trial (1997), which explored the impact of digoxin on outcomes in 6,800 HF patients with LVEF ≤45% and sinus rhythm (96). In those randomised to digoxin therapy, there was a trend towards fewer HF hospitalisations when compared to placebo, but no reduction in all-cause mortality, and a trend towards excess CV deaths. Numerous other studies, including a large meta-analysis of 91,379 HF patients, have gone further and demonstrated an independent mortality risk from digoxin in the HF cohort, in both those with or without AF (97–99). Such trials have contributed to a downtrend trend in the prescription of digoxin for HF in recent years (100), but it remains a class I indicated medication for AF rate control in those with LVEF <40%. ESC cite “*selection and prescription bias*” as the likely reason behind observed associations between digoxin use and excess mortality in patients with AF (101). Indeed, because digoxin is more often prescribed in sicker patients as a second or third-line agent, it is prone to distortion in observational trials.

More RCTs exploring the use of digoxin for AF rate control in heart failure are required, but based on the currently available evidence, we would advise some caution in the use of digoxin for arrhythmia management in CRT, especially in those with a prior history of ventricular arrhythmia or those without defibrillators.

## The use of device data in CRT arrhythmia management

Part of the management of arrhythmia in CRT is the reliable *detection* of arrhythmia. We know from studies such as EAST-AFNET 4 that employing an early rhythm control strategy has positive impacts on cardiovascular outcomes (8). Having continuous monitoring from CRT devices enables the earliest possible detection of new-onset arrhythmias, which not only facilitates prompt catheter ablation of symptomatic arrhythmias, but also the detection of *subclinical* arrhythmias. There remains debate regarding the clinical significance and management of subclinical arrhythmia, especially with the exponential rise in the use of wearable monitors amongst the general population. In the CRT population, subclinical atrial arrhythmias in the form of atrial high rate episodes (AHREs) detected on device interrogation have been shown to be associated with increased risk of developing clinical AF and thromboembolic events (102). However, only AHREs of >24 h’ duration have predictive value, with shorter episodes failing to demonstrate a statistically significant association with cardiovascular events (102–104). As a result, the ESC AF guidelines pragmatically suggest considering anticoagulation in those with both a high burden of AHREs and raised CHA_2_DS_2_-VASc score, evaluated on an case-by-case basis (101).

**Figure 7:**
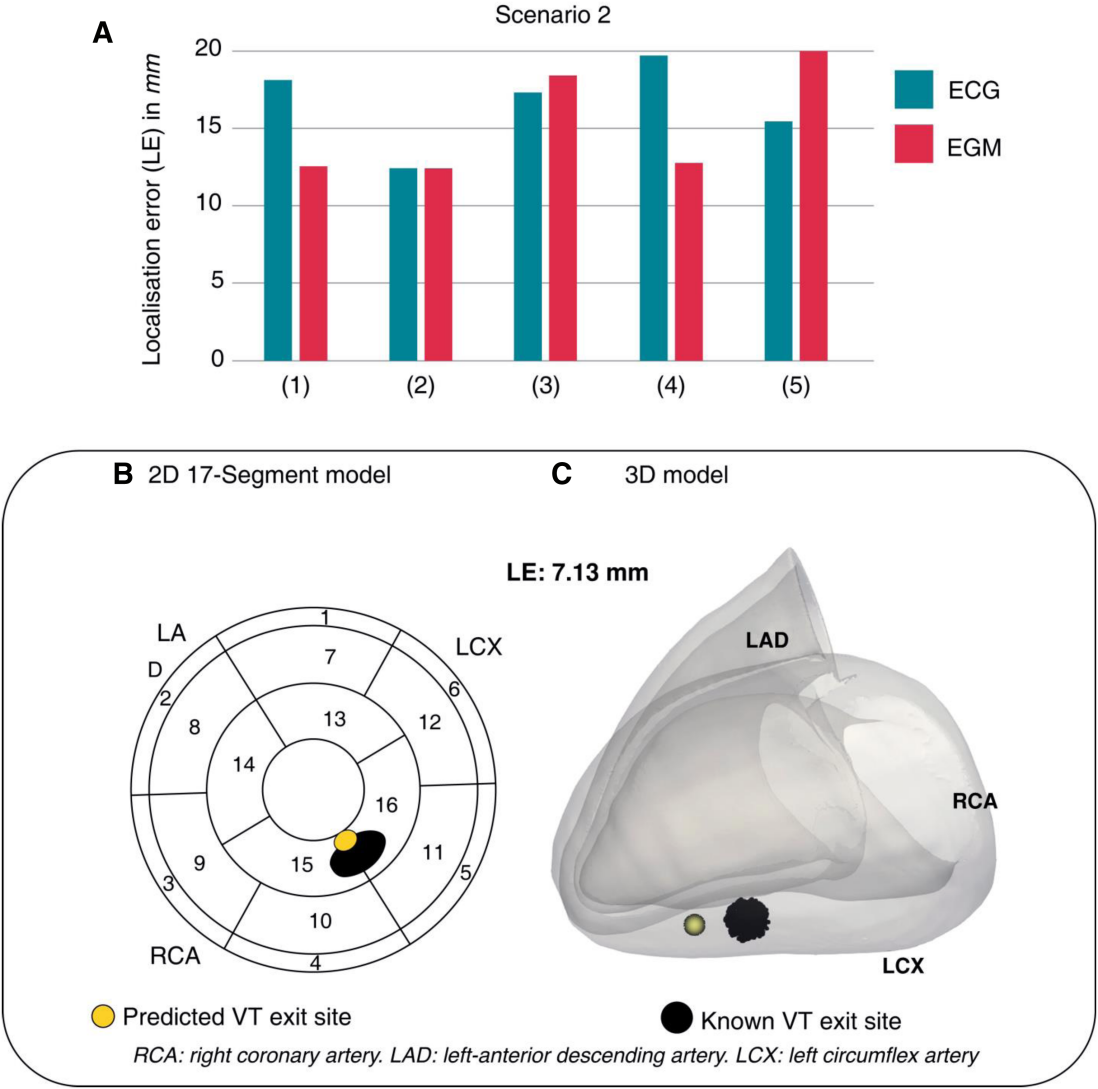
One of Monaci et al.'s computational scenarios. (**A**) LEs across 5 torso models show comparability between ECG and EGM data. (**B,C**) ECG-based prediction of VT exit site (yellow) compared to the known VT exit site (black). LE, localisation error. Reproduced with permission from Monaci et al. (106).

For ventricular arrhythmias, device data has been used to guide ablation planning with novel computational deep learning techniques. This is particularly relevant as the majority of VT ablation referrals are for those with devices already *in situ* (105), and especially exciting for CRT patients, as having an additional LV lead affords further potential for accurate arrhythmia localisation. Monaci et al. utilised device electrogram (EGM) recordings alongside patient CT data to predict the exit-sites of post-infarct VTs (106). They were able to test their computational deep learning framework on real-life patient data, demonstrating accurate prediction of VT exit sites when compared to the known locations in the same patients. Their method of localisation using EGM data performed comparably to ECG-based methods (see Figure 7), illustrating an innovate adoption of deep learning for the management of arrhythmias in device patients. This builds upon the group's previous work, where EGMs derived from CRT-D devices were combined with ECG data to conduct reference-less pace-mapping (107). This particular technique allows for the localisation of VT ablation target sites without any need for inducing or reviewing clinical VT recordings. EGM and ECG data were shown to complement one another, with ECG data providing greater overall spatial coverage and EGMs providing detailed local information due to the proximity of pacing leads to the heart. The specific use of EGMs from CRT-D devices allowed for a particularly rich pool of data, as EGMs were collected from quadripolar LV electrodes in addition to standard dual-coil RV shock leads. The presence of an additional, multipolar pacing lead enhanced the resolution of pace-mapping, demonstrating a unique opportunity for CRT patients with arrhythmia.

EGM-based arrhythmia mapping has several potential advantages over traditional VT mapping. Firstly, it has a strong potential for automation; as research into EGM-mapping progresses, it is plausible that mapping programs could automate VT localisation from hours-worth of device EGM data, and this could be available for use by all VT ablation operators. Indeed, Monaci et al. noted that their reference-less pace-maps can be constructed with relative speed and ease, and thus have the potential to serve as a useful adjunct to existing pace-mapping methods (107). Secondly, it uses pre-collected data, whereas conventional pace-mapping typically requires the induction of VT peri-procedure. Electrical stimulation of VT comes with its own caveats and limitations, such as unclear identification of clinical vs. nonclinical VTs, or non-inducibility of arrhythmia in the electrophysiology (EP) laboratory. Excitingly, EGM-based pace-mapping has been shown to overcome these issues in clinical practice, with Yokokawa et al. using EGMs to localise VT ablation targets in post-infarction patients with otherwise non-inducible VTs (9). They found ICD-EGM-guided ablation was independently associated with a lower risk of VT recurrence (HR: 0.12; 95% CI: 0.03–0.5; *p* = 0.004) during 2 years of follow-up. The utilisation of EGM data to guide catheter ablation is an innovative demonstration of novel techniques and unique opportunities in the management of arrhythmia in the CRT and device population.

## Novel directions

As highlighted throughout, the management of arrhythmia and heart failure is ever evolving, with novel treatments in both fields emerging in recent years. Although beyond the full scope of this article, with more detailed reviews available elsewhere (108), it is impossible to ignore the emergence of conduction system pacing (CSP) as a feasible, theoretically more physiological alternative to traditional BVP. Specifically, the adoption of left bundle branch pacing (LBBP) has provided a solution to the relatively low procedural success rates which hampered its predecessor, His-bundle pacing (HBP) (109).

CSP has been specifically assessed in the management of dyssynchronous heart failure, with promising results thus far. Pujol-Lopez et al. conducted an RCT of CSP vs. BVP in 70 patients indicated for CRT, either for LVEF ≤35% and wide QRS, or AV block and cardiac dysfunction (110). They demonstrated that CSP was noninferior compared to BVP with regards to LV activation time, LV reverse remodelling, NYHA class at 6 months, heart failure hospitalisations or mortality, and QRS shortening. The authors postulate a bias against CSP early in the trial due to the relative learning curve, especially with regards to LBBP, and thus future RCTs may ultimately demonstrate superiority with CSP once familiarity with the procedural technique becomes more universal.

With regards to CSP in the presence of arrhythmia, trials such as Pujol-Lopez et al.'s have included patients with AF, with similar results to sinus rhythm patients. Huang et al.'s observational study of 63 patients with LVEF ≤50% and LBBB demonstrated no significant difference in the beneficial effects of LBBP on LVEF and NYHA functional class between patients with sinus rhythm and those with persistent AF (14/63) (111). CSP has also been demonstrated as a feasible, potentially superior alternative to BVP in persistent AF patients with LVEF ≤40% undergoing AVNA (112). Larger RCTs are needed to explore the efficacy of CSP in patients with all forms of arrhythmia, but all indications thus far are that it will be a viable treatment in this population.

Regarding novel arrhythmia management, stereotactic arrhythmia radioablation (STAR) is emerging as a non-invasive ablation strategy. Its current use is limited to patients with refractory VT in whom catheter ablation has failed or is contraindicated. One case series of 10 patients undergoing STAR observed a 69% reduction in total seconds of detected VT, and a 48% reduction in ATP (113). Although these reductions are relatively modest, patients currently referred for STAR are often amongst the sickest of arrhythmia patients, with the most intractable forms of VT, and co-morbidities which often prohibit catheter ablation. Reassuringly, only one cardiac implantable electrical device (CIED) adverse effect has been reported thus far, involving a rise in RV lead threshold with no further consequence (114), which is important given the vast majority of STAR referrals are for those with ICDs and CRT-Ds *in situ*. STAR is also being considered in the management of refractory AF, but concerns remain about the risk of atrio-oesophageal fistula, and it remains a relatively experimental treatment for this indication.

## Discussion

The subject of arrhythmia management in CRT lacks RCT-level data, with most of our practice being inferred from observational data and larger HF trials. There is no doubt arrhythmia interferes with the ability for CRT to deliver effective BVP, but the specifics of how to (or in the case of AF, whether to even attempt to) achieve rhythm control in CRT patients remain largely untested at an RCT-level. For arrhythmias most suspectable to cure by ablation such as non-AF SVTs, ablation is an attractive option for restoring sinus rhythm and improving CRT response. However, for arrhythmias such as long-standing persistent AF or multifocal PVCs, the treatment strategy is far less straightforward. Patient selection remains a key part of deciding who to put forward for procedural intervention, but modern utilisation of device data has the potential to afford greater opportunities for those with previously poor ablation outcomes, such as those with non-inducible VT. All the while, as the medical management of HF and device technologies continue to advance, so does the relative role of CRT and impact of arrhythmia in the HF population. Ultimately, contemporary RCTs investigating the management of arrhythmia in CRT are sorely needed, for an innovative perspective in the prevailing HF and devices era.
