# Supplementary material for: Managing arrhythmia in cardiac resynchronisation therapy
Source: Front Cardiovasc Med. 2023 Aug 7;10:1211560. doi: 10.3389/fcvm.2023.1211560 (PMC10440957; doi:10.3389/fcvm.2023.1211560)
Supplement: Supplementary file 1 [file Table1.docx]

| **Study (year)** | **Study design** | **Study duration** | **Treatment arms** | **Inclusion criteria** | **% with CRT at baseline** | **N** | **Baseline metrics** | **Median f/u (months)** | **Outcome(s)** | **Results** |
| --- | --- | --- | --- | --- | --- | --- | --- | --- | --- | --- |
| Gasparini et al (2006)  *(AF substudy)* | Multicentre prospective observational | 1995 - 2004 | (A) AVNA if BVP ≤ 85% at 2 months*  (B) OMT  **Amiodarone + Digoxin discontinued at AVNA* | Permanent AF  CRT in situ for:   - LVEF ≤ 35% - NYHA II-IV - QRSd ≥ 120ms   At least 1 HFH 12 months before device implant | 100% | (A) 114  (B) 48 | Age: 66yrs  F*: 14%  LVEF: 26% QRSd: 165ms AAD**: n/a  **% female*  ***% on AADs* | 25 | - LVEF - LVESV - Exercise capacity - CRT response (defined as reduction in LVESV ≥10% from baseline) - All-cause mortality | Improvements in AVNA vs OMT arm in:   - LVEF (p < 0.001) - LVESV (p < 0.001) - Exercise capacity (p < 0.001) - CRT response (68% vs 18%; p = 0.001)   No significant improvement in same parameters in OMT arm from baseline  Higher mortality in OMT arm  (OR 11.1, p < 0.001) |
| Gasparini et al (2008)  *(AF substudy)* | Multicentre prospective observational | 1995 - 2004 | (A) AVNA if BVP ≤ 85% at 2 months*  (B) OMT  **Amiodarone + Digoxin discontinued at AVNA* | Permanent AF  CRT in situ (for any reason) | 100% | (A) 118  (B) 125 | Age: 66yrs  F: 22%  LVEF: 26% QRSd: 162ms  AAD: 54% | 34 | All-cause mortality | Lower mortality in AVNA vs OMT arm (HR 0.31, p = 0.048) |
| PABA-CHF (2008) | Multicentre RCT | 2002 - 2006 | (A) AF ablation  (B) AVNA + de-novo CRTD | Paroxysmal or persistent AF despite AADs  NYHA II or III  LVEF ≤ 40% | n/a | (A) 41  (B) 40 | Age: 61yrs  F: 9%  LVEF: 28%  **QRSd: 91ms** AAD: 100% | n/a  *(100% present at 6mo f/u)* | - LVEF - 6MWD - MLHFQ - Freedom from AF | Greater improvements in AF ablation vs AVNA+CRTD arm in:   - LVEF (+8% vs +1%, p < 0.001) - 6MWD (+71m vs +16m, p <0.001) - MLHFQ (-29 vs -7, p < 0.001) - Freedom from AF (88% vs 0%) |
| Dong et al (2010) | Single centre prospective observational | 2002 - 2006 | (A) AVNA  (B) OMT | CRT in situ for:   - LVEF ≤ 35% - NYHA II-IV despite OMT - QRSd ≥ 120ms | 100% | (A) 45  (B) 109 | Age: 70yrs  F: 15%  LVEF: 25%  QRSd: 168ms  AAD: 19% | 25 | - NYHA - LVEF - LVEDD - All-cause mortality | Greater improvements in AVNA vs OMT arm in:   - NYHA class (p = 0.004) - Survival at 2 years (HR 0.13, p = 0.007)   Comparable improvements in both AVNA and OMT arms in:   - LVEF (8.1% vs 6.8% p = 0.49) - LVEDD (−2.1mm vs −2.1mm, p = 0.74) |
| CERTIFY (2013)  *(AF substudy)* | Multicentre prospective observational | 1999 - 2011 | (A) AVNA  (B) OMT | Permanent AF  CRT in situ for:   - LVEF ≤ 35% - NYHA II-IV - QRSd ≥ 120ms | 100% | (A) 443  (B) 895 | Age: 69yrs  F: 16%  LVEF: 27% QRSd: 157ms AAD use: 29% | 37 | - All-cause mortality - Cardiac mortality - LVEF | Lower all-cause mortality (HR 0.67, p < 0.001) and cardiac mortality (HR 0.63, p < 0.003) in AVNA vs OMT arm  Greater improvement in AVNA vs OMT arm in LVEF at 6 months (+8% vs +4%; p < 0.001) |
| ARC-HF (2013) | Single centre RCT | 2009 - 2012 | (A) AF ablation  (B) Medical rate control | Persistent AF (> 7 days)  NYHA II-IV  LVEF ≤35% | (A) 31%  (B) 12% | (A) 26  (B) 26 | Age: 63yrs  F: 23%  LVEF: 24% QRSd: 116ms *(non-paced only)* AAD: 12% | n/a  *(98% present at 12mo f/u)* | - Peak VO2 - MLHFQ - LVEF - 6MWD - BNP | Improvement in ablation arm vs minor reduction in drug arm in peak VO_2_ (+3.07ml/kg/min difference between arms, p = 0.018)  Greater improvement in ablation vs drug arm in:   - MLHFQ (-21 vs -8, p = 0.019) - BNP (-124ng/L vs -18ng/L, p = 0.045)   Greater, but nonsignificant improvement in ablation vs drug arm in LVEF (+10.9% vs +5.4%, p = 0.055)  No significant difference in 6MWD |
| AATAC-AF (2016) | Multicentre RCT | *Not specified* | (A) AF ablation  (B) AAD *(Amiodarone)* | Persistent AF  ICD or CRTD in situ  NYHA II or III  LVEF ≤40% | 100% with either ICD or CRTD  *(% with CRT not specified)* | (A) 102  (B) 101 | Age: 61yrs  F: 26%  LVEF: 30% QRSd: n/a  AAD:  (A) 0% (B) 100% | No patients lost to f/u at end of 24 month study period  *(13% mortality overall)* | - All-cause mortality - Unplanned hospitalisations - LVEF - MLHFQ - 6MWD - AF burden | Greater improvement in ablation vs AAD arm in:   - LVEF (+8.1% vs +6.2%, p = 0.02) - MLHFQ (-14 vs -2.9, p <0.001) - 6MWD (+27m vs 8m, p <0.001)   AF more likely to recur in AAD vs ablation arm (HR 2.5, p <0.001)  Lower mortality in ablation vs AAD arm (8% vs 18%, p = 0.037)  Lower unplanned hospitalisation rate in ablation vs AAD arm (31% vs 57%, p <0.001) |
| Gasparini et al (2018) | Multicentre prospective observational *(pooled analysis from 2 RCTs and 1 observational trial)* | 2004 - 2014 | (A) AVNA if BVP ≤ 95% at 3 months  (B) OMT | Permanent AF  CRT in situ for:   - LVEF ≤ 35% - NYHA II-IV - QRSd ≥ 120ms | 100% | (A) 262  (B) 402 | Age: 69yrs  F: 15%  LVEF: 27% QRSd: 142ms AAD: 21% | 18 | - ICD shocks (appropriate and inappropriate) - All-cause hospitalisations | Large reductions in AVNA arm vs OMT arm in:   - Appropriate shocks (IRR 0.23, p <0.001) - Inappropriate shocks (IRR 0.09, p <0.001)   Lower all-cause hospitalisations in AVNA arm (IRR 0.57, p < 0.001) |
| CASTLE-AF (2018) | Multicentre RCT | 2008 - 2016 | (A) AF ablation  (B) OMT | Paroxysmal or persistent AF  NYHA II-IV  LVEF ≤35%  ICD or CRTD  Either no response to, unacceptable SEs from or unwilling to take AADs | 28% | (A) 179  (B) 184 | Age: 64yrs  F: 15%  LVEF: 32% QRSd: n/a  AAD: 59%*  *(with either no response to AADs or intolerable SEs)* | 38 | - All-cause mortality - Cardiac mortality - HFH | Lower chance in ablation vs OMT arm of:   - All-cause mortality (HR 0.53, p = 0.009) - Cardiac mortality (HR 0.49, p = 0.008) - HFH (HR 0.56, p = 0.004) |
| AMICA trial (2019) | Multicentre RCT | 2008 - 2017 | (A) AF ablation  (B) OMT +/- DCCV | Symptomatic or longstanding (1-4 yrs) persistent AF  NYHA II or III  Indicated for ICD or CRTD  LVEF ≤35%  LAd <60mm | 24% at enrolment  44% at discharge | (A) 68  (B) 72 | Age: 65yrs  F: 10%  LVEF: 27%  QRSd: n/a  AAD: 32% | 12 | - All-cause mortality - SAEs - LVEF - MLHFQ - 6MWD - NT-pro BNP - AF burden | Comparable improvements in ablation and OMT arms in:   - LVEF (+8.8% vs +7.3%, p = 0.36) - MLHFQ (-11.2 vs -8.9, p = 0.42) - 6MWD (+46m vs +81m, p = 0.07) - NT-proBNP (-891ng/L vs - 419ng/L, p = 0.60)   No differences between arms in mortality (8.2% vs 8.0%) or proportion of patients with ≥1 SAE (65% vs 56%, p = 0.19) |
| Fink et al (2019) | Single centre retrospective observational | 2010 - 2017 | AF ablation | CRT nonresponders, defined as ≥ 1 of:   - BVP <95% due to AF - <1 point ΔNYHA - ≤5% ΔLVEF | 100% | 38 | Age: 68yrs  F: 21%  LVEF: 30% QRSd: n/a  AAD: 71% | 27 | - Freedom from AF - BVP - LVEF - NYHA | 67% freedom from AF at 2 yrs  Improvements from baseline in:   - BVP (+8% p< 0.001) - LVEF (+2.2% p = 0.023) - NYHA class (p < 0.0001) |
| RAFT-AF (2022) | Multicentre RCT | 2011 - 2018 | (A) AF ablation  (B) OMT +/- AVNA if needed  *(% undergoing AVNA not specified)* | NYHA II or III despite OMT    Elevated BNP    High burden pAF or persistent AF < 3 yrs  LAd <55mm | 13% | (A) 214  (B) 197 | Age: 67yrs  F: 26%  LVEF: 40% QRSd: n/a  AAD: 42% | 37 | - All-cause mortality - HF event - MLHFQ - 6MWD - NT-pro BNP - LVEF | Non-significant trends in ablation vs OMT arm towards lower:   - All-cause mortality (HR 0.71, p = 0.066) - HF events (HR 0.79, p = 0.349)   Greater improvements in ablation vs OMT arm at 2 yrs in:   - MLHFQ (p = 0.0036) - 6MWD (p = 0.025) - NT-proBNP (p < 0.0001) - LVEF (+10.1% vs + 3.8%, p = 0.017) |
| APAF-CRT (2021) | Multicentre RCT | 2014 - 2020 | (A) AVNA + de-novo CRT  (B) OMT | Severely symptomatic permanent AF where AF ablation had failed or deemed unsuitable  QRS ≤ 110ms  ≥ 1 HFH in last yr | n/a | (A) 63  (B) 70 | Age: 73yrs  F: 36%  LVEF: 41% QRSd: 95ms AAD: 6% | 29 | - All-cause mortality - HFH | Lower all-cause mortality in AVNA+CRT vs OMT arm (HR 0.26, p = 0.004), but   - only significant when stratified for LVEF >35% (HR 0.27, p = 0.02) - nonsignificant in pts with LVEF ≤35% (HR 0.34, p = 0.22)   Lower rate of combined endpoint of all-cause mortality or HFH in AVNA+CRT arm (HR 0.40; p = 0.002) |

**Table 1: Summary of AF ablation and AVNA studies in the CRT population**

6MWD = 6-minute walk distance; AAD = anti-arrhythmic drug; AF = atrial fibrillation; AVNA = atrioventricular node ablation; BNP = B-type natriuretic peptide; BVP = biventricular pacing; CRT = cardiac resynchronisation therapy; CRTD = cardiac resynchronisation therapy with defibrillator; DCCV = direct current cardioversion; f/u = follow-up; HF = heart failure; HFH = heart failure hospitalisation; HR = hazard ratio; ICD = implantable cardioverter-defibrillator; IRR = incidence rate ratio; LAd = left atrial diameter; LVEDD = left ventricular end-diastolic diameter; LVEF = left ventricular ejection fraction; LVESV = left ventricular end-systolic volume; MLHFQ = Minnesota living with heart failure questionnaire; NT-proBNP = N-terminal pro B-type natriuretic peptide; NYHA = New York Heart Association; OMT = optimal medical therapy; OR = odds ratio; QRSd = QRS duration; RCT = randomised controlled trial; SAEs = serious adverse events; SEs = side effects; VO_2_ = peak oxygen consumption
